# Assessing health and economic outcomes of interventions to reduce pregnancy-related mortality in Nigeria

**DOI:** 10.1186/1471-2458-12-786

**Published:** 2012-09-14

**Authors:** Daniel O Erim, Stephen C Resch, Sue J Goldie

**Affiliations:** 1 Center for Health Decision Science, Harvard School of Public Health, 718 Huntington Avenue, 2nd floor, Boston, MA 02115, USA; 2 Harvard Global Health Institute, Harvard University, Cambridge, MA, USA; 3 Harvard University Center for Geographic Analysis, Cambridge, MA, USA

## Abstract

**Background:**

Women in Nigeria face some of the highest maternal mortality risks in the world. We explore the benefits and cost-effectiveness of individual and integrated packages of interventions to prevent pregnancy-related deaths.

**Methods:**

We adapt a previously validated maternal mortality model to Nigeria. Model outcomes included clinical events, population measures, costs, and cost-effectiveness ratios. Separate models were adapted to Southwest and Northeast zones using survey-based data. Strategies consisted of improving coverage of effective interventions, and could include improved logistics.

**Results:**

Increasing family planning was the most effective individual intervention to reduce pregnancy-related mortality, was cost saving in the Southwest zone and cost-effective elsewhere, and prevented nearly 1 in 5 abortion-related deaths. However, with a singular focus on family planning and safe abortion, mortality reduction would plateau below MDG 5. Strategies that could prevent 4 out of 5 maternal deaths included an integrated and stepwise approach that includes increased skilled deliveries, facility births, access to antenatal/postpartum care, improved recognition of referral need, transport, and availability quality of EmOC in addition to family planning and safe abortion. The economic benefits of these strategies ranged from being cost-saving to having incremental cost-effectiveness ratios less than $500 per YLS, well below Nigeria’s per capita GDP.

**Conclusions:**

Early intensive efforts to improve family planning and control of fertility choices, accompanied by a stepwise effort to scale-up capacity for integrated maternal health services over several years, will save lives and provide equal or greater value than many public health interventions we consider among the most cost-effective (e.g., childhood immunization).

## Background

Nigeria accounts for 1 in 6 maternal deaths globally. Approximately 50,000 Nigerian women die each year from largely preventable pregnancy-related complications [1,2]. With a maternal mortality ratio (MMR) estimated at 840 per 100,000 live births, each of the 34 million women in their reproductive years face a 1 in 23 lifetime risk of maternal death [1-4]. In recognition of this, the Nigerian government and its partners have put in considerable efforts to reduce this burden in line with MDG 5 (e.g. providing free healthcare services to pregnant women, deploying over 4,000 midwives to areas of greatest need, distributing free contraceptive products etc [5,6]). Despite these efforts, the challenges are formidable and progress towards MDG 5 has been below expectations [7-9].

There is little debate about the need for an adequate supply of skilled birth attendants, functional referral systems, reliable transport, and well-equipped facilities [9,10]. However, there is little guidance about how to adapt ideal recommendations to local situations, decide where initial efforts should be targeted, and design an effective and efficient plan to scale-up maternal health services [6,10]. This is further compounded by the regional variations in maternal indices arising from unequal distribution of healthcare infrastructure and manpower (e.g. the MMR in the Southwest and Northeast zones are 165 and 1,549 per 100,000 live births respectively [9]).

With increasing attention from the Nigerian government [5] and other stakeholders [11], this is an opportune moment for deliberative action. To effectively leverage international attention that has catapulted MDG 5 onto the global political agenda, [12] and catalyze efforts being made from within the country, identifying evidence-based strategies that consider the local context is imperative. In this analysis, we synthesize the best available data, adapt a model of pregnancy and pregnancy-related morbidity and mortality to the Nigerian context, and conduct national and regional analyses that quantify the payoffs from investing in safe pregnancy and childbirth. Our purpose is to provide qualitative insight into the most efficient strategies to meet MDG 5.

## Methods

### Overview

Country- and region-specific data were synthesized using a computer-based model that simulates the natural history of pregnancy and childbirth. Separate models were adapted to Southwest and Northeast zones using survey-based data and information about recognition of the need for referral, access to transport, and appropriate facilities [8,9,13]. Model outcomes include clinical events (e.g., pregnancies, live births, maternal complications), measures of maternal mortality (e.g., MMR, proportionate mortality ratio [i.e., proportion of deaths among women aged 15–45 that are pregnancy-related], and lifetime risk of maternal death), population outcomes (e.g., life-expectancy), and costs.

Strategies consisted of increasing the coverage of effective interventions, and could include improved logistics. Following standard recommendations for economic evaluation, we calculated incremental cost-effectiveness ratios, defined as the additional cost of a specific strategy divided by its additional clinical benefit, compared with the next least expensive strategy [14]. We considered interventions with cost-effectiveness ratios of less than the per capita GDP ($1,170) to be very cost-effective [2,15]. Sensitivity analyses are conducted to assess the impact of parameter uncertainty, and Monte Carlo simulation was used to generate the number of per woman events such as pregnancies, live births, and facility-based births.

### Model

The Global Maternal Health Policy Model is a previously published computer-based model that simulates the natural history of pregnancy and pregnancy-related complications over a woman’s lifetime, and aggregates outcomes to a population level [16]. Factors modeled at the individual level include the probability of pregnancy (conditional on age, contraceptive use, and clinical history), the probability of spontaneous or induced abortion, and the risk of direct pregnancy-related complications such as hypertensive disorders, obstructed labor, hemorrhage, and sepsis. The case fatality rates of these complications are conditional on the type, severity and underlying comorbidity. Nonfatal secondary complications considered include neurological sequelae, obstetric fistula, severe anemia, and infertility. In addition to pregnancy-related mortality risk, women face an annual risk of death from age-specific all-cause mortality. The model is described in more detail in the Additional file 1.

Strategies to reduce maternal deaths consist of improving coverage of effective interventions, either individually or packaged as integrated services. These include: reducing the unmet need for contraception; increased accessibility to safe abortion and post-abortion care; prevention and treatment of anemia (including intermittent prevention and treatment of malaria in pregnancy); and increased availability of intrapartum and postpartum care. Recognizing that the investments in infrastructure required to assure high-quality intrapartum care will need to happen in phases, stepwise improvements are modeled over time.

**Figure 1:**
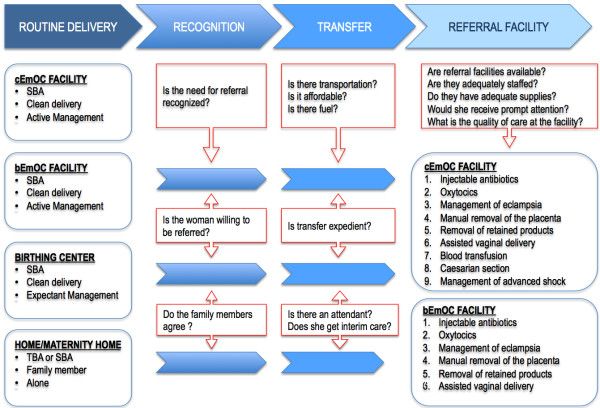
**Critical delays experienced during labor and delivery.** The intervention path during labor and delivery (as contained in the model) shows the location, attendant, and three potential barriers to effective treatment in the event of a complication, including recognition of referral need, transfer (e.g., transport), and timely quality care in an appropriate EmOC facility [16].

The overall impact of interventions results from a reduction in the incidence and/or case fatality rate of a complication. Both mechanisms depend, in part, on access to specific services, trained personnel, and quality of the facilities delivering these services. Therefore, the model explicitly considers the location of delivery, type of assistance, access to basic or comprehensive obstetrical care, and the ability to overcome barriers around the timing of delivery (See Figure 1). Delivery setting is differentiated by provider (e.g., family member, traditional birth attendant [TBA], SBA or no one) and by site (e.g., home versus facility). Facilities providing basic EmOC (bEmOC) are assumed to be capable of administering injectable antibiotics, oxytocic drugs, and sedatives or anti-convulsants, and also conducting assisted vaginal delivery, removal of placenta and retained products. Facilities capable of comprehensive EmOC (cEmOC) are able to provide blood transfusion services, cesarean delivery, and management of advanced shock in addition to all the aforementioned bEmOC services [17].

Health facilities in Nigeria are classified as primary, secondary and tertiary [7]. Primary care facilities (e.g. Alausa Primary Health Care center, Lagos State) are the most abundant, but SBAs aren’t always available, and only few can provide all the services that constitute bEmOC [18,19]. Conversely, all tertiary facilities (e.g. University College Hospital Ibadan, Oyo State), and most secondary facilities (e.g. General Hospital Calabar, Cross River State) can provide comprehensive emergency obstetric care, but they are fewer, and are predominantly in urban areas [19]. In adapting the model to the Nigerian context, we map the current facility structure on to the model structure described above.

The model is programmed using TreeAge Pro 2009 (TreeAge Software Inc., Williamstown MA) and analyses conducted using Microsoft Excel 2010 and Visual Basic for Applications 7 (Microsoft Corp., Redmond WA).

### Data and assumptions

Selected model inputs and assumptions are shown in Table 1 and Table 2. In general, estimates of incidence and case fatality rates associated with pregnancy-related complications were obtained from published data, and a plausible range for sensitivity analysis was based on systematic review of the literature. Data on facility births, skilled birth attendants, family planning, and antenatal care were from country-specific surveys and government reports (more details in the Additional file 1).

**Table 1:** Selected data and assumptions on parameters used in the model (Coverage and costs)

**Coverage**	**Baseline (%) [**[13]**]**
Current use of family planning	
·Any method	14.6
·Modern methods	9.7
·Unmet need	20.2
Antenatal care coverage	57.7
Treatment of anemia [20]	54.3
Total skilled delivery	38.9
Facility delivery	35.0
Home delivery with a skilled birth attendant	6.9
**Estimates of costs under current standard of care (*2008 US$*)**	**Model input [**[21]**]**
Family planning	
·Oral contraceptives	$13.54
·Injectable contraceptives	$13.51
·Condoms	$11.30
·Intrauterine device	$13.31
·Female sterilization	$23.29
·Male sterilization	$16.46
Pregnancy and delivery or abortion.	
·Antenatal care (four visits) ^a^	$23.75
·Anemia treatment (based on severity)	$0.68-1.02
·Abortion	
−Post-abortion complications	$50.73
−Elective abortion	$21.87
Delivery ^b^	
·Home (TBA; SBA)	$7.99; $11.53
·Facility (birthing center; bEmOC; cEmOC)	$20.59; $35.00; $46.33
Postpartum care (one visit) ^c^	$7.14
Transportation ^d^	$5.15 - $11.58
Management of complications ^e^	
·Obstructed labor	$23.63 - $109.96
·Maternal hemorrhage	$34.71 - $150.78
·Puerperal sepsis	$39.08 - $83.90
·Severe pre-eclampsia/eclampsia	$73.82 - $116.17

Costs estimates were obtained from the UNFPA Reproductive Health Costing Model (RHTCM) [21], and WHO CHOICE public databases [22]. bEmOC = Basic Emergency Obstetric Care; cEmOC = Comprehensive Emergency Obstetric Care; TBA = Traditional Birth Attendant; and SBA = Skilled Birth Attendant. Details and methods for converting to 2008 US$ are provided in the Additional file 1.

^a^ Cost of prenatal care accrue from the following: Drugs (e.g. iron supplements and folic acid, tetanus toxoid, etc.), tests (blood group, hemoglobin, blood glucose, pregnancy test, Rapid Plasma Reagin test [syphilis], HIV test, Urinalysis, etc.), materials needed to safely administer the aforementioned drugs or conduct the tests, and personnel costs (nurse/midwife, Obstetrician and Lab technician) for about 4 antenatal visits [21].

^b^ Total cost reflects the skill of the attendant, level of facility, drugs and supplies. See the Additional file 1 for details.

^c^ Postpartum care includes examination, iron/folate supplement and counseling. The cost of post partum care accrue from drugs (iron supplements and folic acid) and personnel costs (nurse/midwife).

^d^ Transportation costs include those incurred from home to a referral facility (bEmOC or cEmOC), and those incurred between facilities when necessary [23]. See the Additional file 1 for details. The range for transportation costs encompasses cost of transport from home to a health facility (birthing center, bEmOC or cEmOC facility), and between health facilities (birthing center to bEmOC or cEmOC and bEmOC to cEmOC).

^e^ The range of costs to manage complications reflects varying severity levels and whether or not management requires a bEmOC or cEmOC facility.

**Table 2:** Input parameters for direct complications of pregnancy and childbirth

**Direct complications**	**Estimates**	**Range**
**Postpartum hemorrhage (PPH)**		
*Incidence and mortality*		
−PPH, probability of event (range) [24,25]	0.114	(0.051-0.228) ^g^
−PPH, probability of morbidity (range) ^b^[26-29]	0.008	(0.006-0.010)
−PPH, case fatality rate (CFR) [30-32]	0.010	
−PPH, adjusted CFR (range) ^a^	0.023	(0.007-0.030) ^g^
*Impact of interventions*		
−PPH, decrease in incidence (range) [33,34]^c, d^	50%, 75% ^g^	(25%-91%)
−PPH, decrease in case fatality rate (range) ^d, e^[30,35-41]	75%	(60% - 90%) ^g^
**Obstructed labor (OL)**		
*Incidence and mortality*		
−OL, probability of event (range) [24,25]	0.047	(0.030-0.074) ^g^
−OL, probability of morbidity (range) ^b^[26-29]	0.022	(0.018-0.026)
−OL, case fatality rate (CFR) [30-32]	0.007	
−OL, adjusted CFR (range) ^a^	0.019	(0.005-0.025) ^g^
*Impact of interventions*		
−OL, decrease in incidence (range) [33,34]^d^	-	
−OL, decrease in case fatality rate (range) ^d, e^[30,35-41]	95%	(76% - 100%) ^g^
**Hypertensive disorders of pregnancy (HD)**		
*Incidence and mortality*		
−HD, probability of event (range) [24,25]	0.035	(0.025-0.05) ^g^
−HD, probability of morbidity (range) ^b^[26-29]	0.001	(0.001-0.001)
−HD, case fatality rate (CFR) [30-32]	0.017	
−HD, adjusted CFR (range) ^a^	0.021	(0.012-0.027) ^g^
*Impact of interventions*		
−HD, decrease in incidence (range) [33,34]^d^	NA	(25%-50%)
−HD, decrease in case fatality rate (range) ^d, e^[30,35-41]	59%	(45% - 95%) ^g^
**Sepsis**		
*Incidence and mortality*		
−Probability of event (range) [24,25]	0.050	(0.043-0.060) ^g^
−Probability of morbidity (range) ^b^[26-29]	0.400	(0.320-0.480)
−Case fatality rate (CFR) [30-32]	0.013	
−Adjusted CFR (range) ^a^	0.028	(0.009-0.036) ^g^
*Impact of interventions*		
−Decrease in incidence (range) [33,34]^c, d^	25%, 50%	(0%-60%)
−Decrease in case fatality rate (range) ^d, e^[30,35-41]	90%	(63% - 93%) ^g^
**Unsafe abortion (UA)**		
*Incidence and mortality*		
−UA, probability of event (range) ^f^[24,25]	0.128	(0.050-0.250)
−UA, probability of morbidity (range) ^b^[26-29]	0.120	(0.096-0.144)
−UA, case fatality rate (CFR) [30-32]	0.003	
−UA, adjusted CFR (range) ^a^	0.009	(0.002-0.012)
*Impact of interventions*		
−UA, decrease in incidence (range) [33,34]^d^	NA	(0%-100%)
−UA, decrease in case fatality rate (range) ^d, e^[30,35-41]	98% ^c^	(50% - 100%)

^a^ CFRs were adjusted based on complication severity (e.g., life threatening complications requiring cEmOC) and underlying severity of anemia [42].

^b^ Examples of nonfatal complications include Sheehan’s syndrome from obstetric hemorrhage, fistula from obstructed labor, neurologic sequelae from eclampsia, pelvic inflammatory disease (PID).Not shown but included are the risk of infertility from PID (0.086), and the risk of severe anemia following obstetric hemorrhage (0.09) [27,43].

^c^ The incidence of sepsis reduced by 50% with SBA and clean delivery in birthing center, bEmOC, and cEmOC; and reduced by 25% with SBA and clean delivery at home [34]. Incidence of maternal hemorrhage reduced by 50%–75% depending on expectant versus active management of labor; we assume for the status quo, all cEmOC facilities provide active management, 50% of bEmOC facilities provide active management, and birthing centers/health centers provide expectant management only [33].

^d^ For each baseline estimate, sensitivity analysis was conducted across a plausible range based on literature review; references and assumptions are documented in the Additional file 1.

^e^ Estimates shown represent average reduction in case fatality rate provided complications necessitating surgery (e.g., cesarean section), blood transfusion, intensive hemodynamic support are treated in cEmOC. Obstructed labor is managed using assisted vaginal delivery with forceps or vacuum and, if necessary, cesarean section; severe pre-eclampsia and eclampsia treated with intravenous hydralazine and magnesium sulfate, in addition to induction of labor or emergency cesarean section when required; sepsis treated with ampicillin, gentamycin, and metronidazole or equivalent regimen followed by an 8-d course of intramuscular gentamycin and oral metronidazole (see Additional file 1 for details) [30,44].

^f^ Incidence of elective abortion is 0.170, all of which are assumed to be unsafe in the base case. Case fatality rate (CFR) of safe abortion is 0.000006; representing a 98% reduction in mortality (see Text S1). Incidence of miscarriage (not shown) is 0.150 [25].

^g^ These ranges were used to assess parameter uncertainty on the incidence and CFR of direct maternal complications, and effectiveness of interventions (see Table 6).

For women delivering outside an EmOC facility, the probability of a successful referral depended on overcoming three categories of delays: delay in recognizing need for referral and being willing to go; delay in transport to referral facility; and delay in receiving appropriate care at appropriate EmOC facility. Assumptions about barriers to successful referral were based on country reports, published and grey literature, as well as in-country visits (between March and December 2010) to elicit expert local opinion [19,45-48]. We conducted an in-country survey of 121 healthcare facilities and 700 women aged 15–45 (see Additional file 1) to provide insight into the range of values for sensitivity analysis [49]. After using the best available data to parameterize the national and sub-national models (for Southwest and Northeast zones), key outcomes were generated and compared to independent data. Selected results of this exercise are shown in Table 3, and the procedure used is described in the Additional file 1.

**Table 3:** Model validation (some model outputs being compared to published estimates)

**Maternal indices (National)**	**Estimates**	**Model output**
*Maternal mortality rate (per 100,000 live births)*		
·Published estimates from WHO [50]	800	800
·Published estimates from World Bank [2,51]	840	800
*Total fertility rate*		
·Published estimates from WHO, World Bank and Nigeria DHS 2008 [2,13,50]	5.7	5.8
*Annual number of maternal deaths (modeled estimate)*		
·Published estimates from WHO [51]	50,000	53,000
**Maternal indices (zonal)**	**Estimates**	**Model output**
*Maternal mortality rate (per 100,000 live births)*		
·Southwest	165 [9]	170
·Northeast	1,549 [9]	1,557
*Total fertility rate*		
·Southwest	4.5 [13]	4.6
·*Northeast*	7.2 [13]	7.2

Selected key outcomes from the model being compared to independent data.

### Cost inputs

Selected costs are shown in Table 1. Details of the costing methodology are included in the Additional file 1. Costs of delivering interventions and treating maternal complications were estimated from the United Nations Population Fund’s (UNPF) Reproductive Health Costing Tools (RHCT) [21]. Costs associated with personnel (salaries) were from public access databases [52,53]. Drugs and supply costs were from the United Nations Children Fund’s (UNICEF) Supply Catalogue [54] and Management Sciences for Health (MSH) International Drug Price Indicator Guide [54,55]. To estimate the costs of improving transport and scaling up facilities we used methods previously described [16] and assumptions from in-country experts. All costs were converted to 2008 U.S. dollars.

## Results

### Reducing the unmet need for fertility control (preventing, spacing and limiting births)

Modern contraceptive prevalence rates range from 3.5% in the Northeast zone (17.6% unmet need) to 21% in the Southwest zone (19.7% unmet need) [8,9,13]. Reducing the unmet need by 25% to 100% reduced maternal deaths by 4% to 17% in the Southwest and 3% to 13% in the Northeast zone (Table 4, see Additional file 1). Because the unmet need is based on survey-derived preferences of Nigerian women questioned *now,* and the number of desired children remains high, modeling elimination of the unmet need only reduces the TFR from 5.9 to 4.9. Anticipating changes to fertility preferences over time, we conducted a secondary analysis in which the use of modern contraceptives was increased by 25% to 50%, corresponding to a contraceptive prevalence rate of 34.7% and 59.7%, respectively (Table 4). The TFR was reduced from 5.9 to 4.4 (25% increase), and to 2.9 (50% increase). With a contraceptive prevalence rate of 59.7%, approximately 1 out of 2 maternal deaths was prevented. All of the above strategies were extremely cost-effective with incremental cost-effectiveness ratios less than $10 per year of life saved. Additionally, increasing the use of modern contraceptives in the model led to a decline in abortion related deaths (more details in the Additional file 1).

**Table 4:** Changes in maternal health indices, predicted averted deaths and associated costs (or savings) that accompanied a stepwise reduction in the unmet need for contraception

**Maternal Health Index**	**Status quo**	**Primary analysis on benefits of family planning**				**Secondary analysis on benefits of family planning**			
		**Reducing the unmet need for contraception**				**Increasing use of modern contraceptive method**			
		**by 25%**	**by 50%**	**by 75%**	**by 100%**	**by 25%**	**by 30%**	**by 40%**	**by 50%**
**National analysis**									
Prevalence of modern methods of contraception (average) ^a^	9.7%	14.8%	19.8%	24.9%	29.9%	34.7%	39.7%	49.7%	59.7%
Reduction in maternal deaths (%)	-	5.9%	9.7%	13.5%	17.4%	24.2%	29.1%	39.1%	49.9%
Total fertility rates	5.90	5.60	5.30	5.10	4.90	4.40	4.11	3.55	2.96
Lifetime risk of maternal deaths	1 in 26	1 in 28	1 in 29	1 in 31	1 in 32	1 in 36	1 in 38	1 in 44	1 in 53
Proportionate mortality risk	14%	13%	12%	12%	11%	10%	10%	8%	7%
Maternal deaths averted per 100,000	-	221	367	512	659	897	1,080	1,449	1,814
Additional costs per woman over lifetime (US$)	$0.00	$2.46	$4.81	$7.18	$9.55	$16.50	$19.84	$26.55	$33.30
Additional cost to cohort over lifetime (million US$) ^b^	-	$85.26	$166.89	$248.81	$331.02	$527.12	$687.92	$920.56	$1,154.59
Cost effectiveness ratio (rounding) (US$ per YLS)	-	6.40/YLS	6.50/YLS	6.60/YLS	6.70/YLS	6.90/YLS	7.10/YLS	7.30/YLS	7.60/YLS
**Zonal analysis (Southwest zone)**									
Prevalence of modern methods of contraception (average) ^a^	21.0%	24.6%	28.1%	31.7%	35.2%	46.0%	51.0%	61.0%	71.0%
Reduction in maternal deaths (%)	-	4.3%	8.5%	12.9%	17.1%	30.2%	36.3%	48.5%	60.8%
Total fertility rates	4.60	4.40	4.20	4.00	3.80	3.20	2.92	2.36	1.80
Lifetime risk of maternal deaths	1 in 128	1 in 134	1 in 140	1 in 147	1 in 155	1 in 184	1 in 201	1 in 249	1 in 328
Proportionate mortality risk	3.1%	2.9%	2.8%	2.7%	2.6%	2.2%	2.0%	1.6%	1.2%
Maternal deaths averted per 100,000	-	34	67	100	133	236	283	378	474
Additional costs per woman over lifetime (US$)	$0.00	-$0.72	-$1.44	-$2.18	-$2.90	-$5.13	-$6.17	-$8.26	-$10.36
Additional cost to cohort over lifetime (million US$) ^b^	-	-$4.49	-$9.00	-$13.65	-$18.18	-$32.20	-$38.72	-$51.80	-$64.96
Cost effectiveness ratio (rounding) (US$ per YLS)	-	8.20/YLS	8.10/YLS	8.10/YLS	8.10/YLS	8.00/YLS	7.90/YLS	7.80/YLS	7.70/YLS

Each step is in comparison with current conditions. YLS = Year or life saved.

^a^ In the model, age specific rates for use of contraception [13] were used in the national analysis, hence the average values are presented above. For the zonal analysis, average contraceptive rates were used and are presented above.

^b^ Cohort here is made up of the estimated number of women aged 15–45 years old. This amounts to 34.67 million nationally, 6.27 million in Southwest zone, and 3.95 million in the Northeast zone. National figures were derived from the UN World Population Projections [56], and the zonal figures from the 2006 census (here, the estimated proportion of women aged 15–45 years in each zone [18.5% and 11.7% in the Southwest and Northeast respectively] were applied to the UN World Population Projection). Additionally, negative costs imply cost savings. Results from the Northeast zonal analysis are in the supplemental text.

### Interventions packaged as integrated services

**Figure 2:**
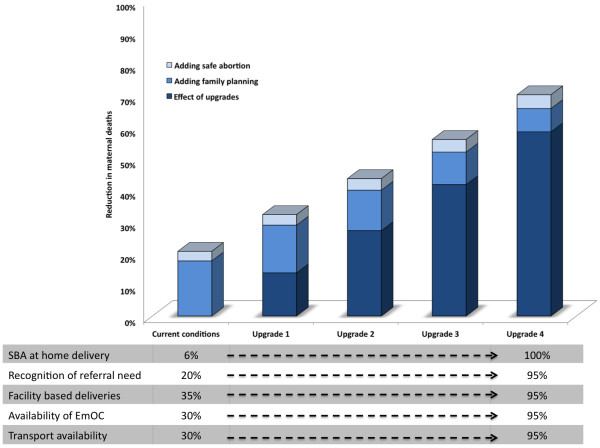
**Reduction in maternal deaths - incremental benefits of upgrades, family planning and safe abortion.** The effect of combining upgrades, safe abortion and family planning on reducing maternal deaths. SBA = skilled birth attendants, and EmOC = emergency obstetric care. “Status quo” refers to the model’s estimate of the current total number of maternal deaths per year (48,480), and an average life expectancy of 47 years (compared to 50,000 and 48 years respectively from the published literature). "**Adding family planning"** means a complete reduction in unmet need for contraception and "**Adding safe abortion"** means universal access to safe abortion services. **"Upgrades”** refers to strategic increments in SBA supervised home deliveries, recognition of need for referral, facility based deliveries and availability of EmOC centers and emergency transportation. These increments are from baseline or "**Status quo**" to 90% -100% (**Upgrade 4**). The height of each stacked column represents the estimated reduction in maternal deaths when **family planning** and **safe abortion** are added to the **upgrades**. However, each color-coded segment represents the contribution from the respective intervention.

Figure 2 shows the benefits expected from improving intrapartum care (upgrades), superimposed with the additional benefits from reducing the unmet need for contraception and increased coverage of safe abortion services. Strategies that improved intrapartum care alone had higher cost-effectiveness ratios (i.e., least attractive), reflecting the higher costs required for infrastructure improvements. Strategies that only improved family planning and safe abortion had very low cost-effectiveness ratios (i.e., very attractive), but reduced mortality by 20.5%. A strategic approach that involves simultaneous improvement in intrapartum care, family planning and safe abortion was the most efficient and associated with incremental cost-effectiveness ratios between the two aforementioned strategies. Further details are provided in the Additional file 1.

Table 5 provides robust insight into the importance of investing in all three domains of family planning, safe abortion and intrapartum care – any approach that focuses on only one of these, to the exclusion of the other, will be less effective and cost-effective. But decision makers will still need to decide on *how* to proceed with such stepwise investments in all three domains. While these choices will be a function of many contextual factors, we sought to provide decision makers with information on the expected benefits, value and efficiency of different approaches. We applied a time dimension of twelve years to the analysis to stacked cohorts, and conducted a stylized exercise to identify scale-up approaches that would be more and less efficient.

**Table 5:** Changes in maternal deaths and incremental costs that could accompany increased coverage of select maternal interventions

	**2011**	**2012**	**2013**	**2014**	**2015**	**2016**	**2017**	**2018**	**2019**	**2020**	**2021**	**2022**	**Total**
**APPROACH 1**													
**Interventions**													
Reduction in unmet need for contraception	25%	50%	75%	100%	100%	100%	100%	100%	100%	100%	100%	100%	
·Increase in coverage of safe abortion	30%	50%	80%	100%	100%	100%	100%	100%	100%	100%	100%	100%	
·Facility upgrade package	-	-	-	-	1	1	2	2	3	3	4	4	
**Incremental cost (millions)**	$3.88	$7.91	$12.16	$16.65	$55.27	$56.73	$100.40	$103.00	$151.47	$155.30	$205.89	$210.90	**$1,079.55**
**Maternal deaths averted**	2,795	4,912	7,374	9,691	16,969	17,394	25,125	25,736	34,777	35,594	46,639	47,690	**274,695**
	**2011**	**2012**	**2013**	**2014**	**2015**	**2016**	**2017**	**2018**	**2019**	**2020**	**2021**	**2022**	**Total**
**APPROACH 2**													
**Interventions**													
Reduction in unmet need for contraception	-	-	-	-	-	-	-	-	25%	50%	75%	100%	
·Increase in coverage of safe abortion	-	-	-	-	-	-	-	-	30%	50%	80%	100%	
·Facility upgrade package	1	1	2	2	3	3	4	4	4	4	4	4	
**Incremental cost (millions)**	$41.18	$42.28	$89.08	$91.44	$143.63	$147.40	$202.16	$207.39	$205.77	$207.76	$209.61	$211.30	**$1,798.99**
**Maternal deaths averted**	7,103	7,284	14,866	15,242	24,183	24,781	35,815	36,686	39,766	42,242	45,124	47,672	**340,765**
	**2011**	**2012**	**2013**	**2014**	**2015**	**2016**	**2017**	**2018**	**2019**	**2020**	**2021**	**2022**	**Total**
**APPROACH 3**													
**Interventions**													
·Reduction in unmet need for contraception	25%	25%	50%	50%	75%	75%	100%	100%	100%	100%	100%	100%	
· Increase in coverage of safe abortion	30%	30%	50%	50%	80%	80%	100%	100%	100%	100%	100%	100%	
·Facility upgrade package	1	1	2	2	3	3	4	4	4	4	4	4	
**Incremental cost (millions)**	$42.87	$44.02	$89.20	$91.57	$138.23	$141.86	$186.97	$191.81	$196.73	$201.69	$206.67	$211.66	**$1,743.26**
**Maternal deaths averted**	9,664	9,915	19,053	19,540	29,980	30,729	42,414	43,453	44,501	45,552	46,604	47,655	**389,060**

Select maternal interventions include increased use of modern contraceptives (through reducing unmet need), and safe abortion services, as well as improving intrapartum care (see text). These interventions are applied in three different ways or “***Approaches***” to stacked cohorts of women aged 15–45 years over a 12 year period (from 2011 to 2022). In **Approach 1**, utilization of modern contraceptives and safe abortion services were gradually maximized prior to improving intrapartum care; in **Approach 2**, improvement in intrapartum care was progressively increased to a maximum before the use of modern contraceptives and safe abortion services were increased; in **Approach 3**, all three interventions were gradually maximized concurrently.

For purposes of generating main themes, and not trying to compare an unlimited number of hypotheticals, we restricted our approaches to three general options:

1) **Approach 1**: reduce the unmet need for contraception and increase access to safe abortion before investing in improvements in intrapartum care;

2) **Approach 2**: improve intrapartum care before reducing the unmet need for contraception and increasing access to safe abortion;

3) **Approach 3**: reduce the unmet need for contraception and increase access to safe abortion while also investing in improving intrapartum care.

We used UN population projections for Nigerian women aged 15–45 years over a 15-year period (from 2006 – 2022) and model projected estimates of annual probability of mortality, proportionate mortality ratio and cost [56]. Upon applying data specific for “current status” to the cohort for 2006 and 2007, our population projections approximated UN projections (with age-specific variations in population ranging between −3% and 1% of UN data), as did our estimate of the number of maternal deaths each year (48,483 and 49,810 in 2007 and 2008 respectively). Further details about these methods are provided in the Additional file 1.

We applied model generated probabilities of mortality and costs to the three approaches described above, scaling the interventions over a 12-year period (from 2011 to 2022), and estimated the number of maternal deaths averted with each approach. Approach 1 could avert over 270,000 maternal deaths, while Approach 2 and Approach 3 could respectively avert over 340,000 and 380,000 maternal deaths over 12 year period (see Table 5).

### Sensitivity analyses

Increasing availability of transportation for deliveries referred to EmOC facilities (from 30% to 100%) led to a 1% reduction in maternal deaths, and an ICER that ranged between $2,700 and $15,800 per YLS. Similarly, increasing the recognition of referral need during skilled home deliveries and the quality of care in EmOC facilities in an isolated manner resulted in 3% reduction in maternal deaths. Providing prenatal care to all pregnant women was not an attractive single intervention with only a 3% reduction in maternal deaths (ICER = $3,400 /YLS). However, if it is assumed to increase the odds of a subsequent facility delivery, an additional 5% - 33% of maternal deaths could be averted (ICER < $800 per YLS). We assumed that 30% of facility births occurred in centers that could provide all EmOC services, and that 10% of these occurred in cEmOC facilities. Shifting all routine EmOC deliveries to cEmOC facilities was much less efficient (ICER ranged between $2,000 and $8,500 per YLS) than shifting births to bEmOC centers or birthing centers with reliable attendance and transport to cEmOC centers if needed.

To further assess for uncertainty, we varied several model inputs to limits suggested by empiric evidence (see Table 2). These inputs include the incidence and case fatality rates of direct maternal complications, as well as the effectiveness and cost of maternal interventions (cost were varied between 50% and 100% of the original inputs: see Tables 1 and 2). While varying these inputs, we improved the coverage of effective interventions (i.e. we reduced the unmet need for contraception, increased access to safe abortion and post-abortion care, as well as access to optimal intrapartum and postpartum care). In all instances, the increasing availability of these interventions was cost effective (with ICERs less than $550 per YLS). Additionally, while there were significant differences in outcomes (i.e. the absolute number of maternal deaths, MMR, lifetime risk or maternal death and proportionate mortality risk: see the Additional file 1), the relative impacts of the interventions were constant (except in instances where the effectiveness of the interventions were altered: see Table 6). Nevertheless, the cost-effectiveness (CE) ratios, the ICERs and the predicted reduction in maternal deaths were largely robust (see Table 6).

**Table 6:** Assessing uncertainty of several biological and nonbiological input parameters

**Analysis**	**Predicted reduction in maternal deaths**	**Cost effectiveness ratios (per year of life saved)**
Unchanged parameters (i.e. incidences, CFR and effectiveness)	6% - 65%	6.3 - 10.5
Reduced incidences of direct maternal complications	5% - 62%	6.2 - 9.8
Increased incidence of direct maternal complications	6% - 66%	6.4 - 12.0
Reduced CFR of direct maternal complications	6% - 66%	6.3 -10.5
Increased CFR of direct maternal complications	5% - 65%	6.3 - 10.5
Reduced effectiveness of maternal interventions	5% - 58%	6.3 - 10.5
Increased effectiveness of maternal interventions	7% - 72%	6.3 - 10.5
Reduced costs of maternal interventions	6% - 65%	4.2 – 8.4
Increased costs of maternal interventions	6% - 65%	10.4 – 15.1

Estimates used in the sensitivity analysis are as follows: postpartum hemorrhage [reduced incidence = 0.051; increased incidence = 0.228; reduced CFR = 0.007; increased CFR = 0.03; reduced effectiveness of interventions = 60%; increased effectiveness of interventions = 90%], obstructed labor [reduced incidence = 0.03; increased incidence = 0.074; reduced CFR = 0.005; increased CFR = 0.025; reduced effectiveness of interventions = 76%; increased effectiveness of interventions = 100%], hypertensive disorders of pregnancy [reduced incidence = 0.025; increased incidence = 0.05; reduced CFR = 0.012; increased CFR = 0.027; reduced effectiveness of interventions = 45%; increased effectiveness of interventions = 95%], Sepsis [reduced incidence = 0.043; increased incidence = 0.06; reduced CFR = 0.009; increased CFR = 0.036; reduced effectiveness of interventions = 63%; increased effectiveness of interventions = 93%]. Additional findings are contained in the Additional file 1.

## Discussion

Our principal findings are that early intensive efforts to improve family planning, accompanied by a systematic stepwise scale-up of intrapartum and emergency obstetrical care, could reduce maternal deaths by 75%. Recognizing that a model-based analysis is only as accurate as the quality of the data that are available, and that our data limitations were formidable, there are qualitative insights that appear robust.

First, reducing the unmet need for contraception is the most effective and cost-effective single intervention for reducing maternal deaths in the short term. By simply meeting the total demand for contraception, over 6,500 maternal deaths could be averted each year nationwide. Furthermore, it is cost saving in the Southwest zone (over $18 million in cost-savings), and can provide funds that could be channeled to other zones with greater health needs. This strategy would also prevent 1 in 5 deaths from unsafe abortion [16,57]

Second, there is a threshold above which further reductions in mortality from sole use of contraception are not possible; integrated interventions that couple family planning with reliable access to high-quality intrapartum and emergency obstetrical care are necessary to cross this threshold. Third, even allowing for considerable variation in the pace that would be feasible to scale up maternal health services, strategies that do so by systematically making stepwise improvements in family planning, safe abortion and intrapartum care will be more effective and efficient in the long-run than solely focusing on any one of these alone. A strategy that involved phasic and concurrent improvements in the availability and standard of EmOC facilities, referral systems, access to skilled birth attendants, facility deliveries, availability and use modern contraceptives and access to safe abortion services could prevent three to four out of five maternal deaths. This strategy had cost-effectiveness ratios that were a fraction of Nigeria’s per capita GDP [15].

While our analysis is intended to catalyze actionable steps, we recognize that decisions in Nigeria will involve a number of choices on *how* to proceed with investments to improve maternal health. Since specific approaches will need to be designed to be contextually appropriate for specific settings, we provide generalized results in a matrix (Table 5) that allows policy makers to obtain insight into the predicted benefits expected with a variety of different approaches.

Limitations related to data quality and availability for informing the natural history parameters in addition to the assumptions used to build the underlying model structure have previously been discussed [16]. Data limitations specific to Nigeria are detailed in the Additional file 1. In addition, data were limited for the frequency of unsafe abortion, and estimates of unmet need were based on survey data reflecting women’s desires now, and not in the future. Additionally, data that were available and obtained from previous studies, such as many of the government-sponsored surveys [18,19] each have their own limitations. While these data may be limited in quality, they represent the best information available now. Additionally, the cost inputs are estimates of total cost, and are agnostic about who bears the cost. However, our analysis is from a societal perspective aimed at estimating the total economic (opportunity) cost for the society. The cost outputs represent costs incurred for a cohort (which can also be expressed on a ‘per woman’ basis) over the lifetime of the cohort.

We emphasize that the purpose of this analysis was not to provide precise estimates, but to provide qualitative insight into decisions that will need to be made well before better data become available, and acknowledge the necessity for repeated studies as better data become available. We also acknowledge that other interventions, outside of those included in this analysis, are likely to have major benefits on maternal health through indirect effects (e.g. enactment of policies that improve nutrition and agriculture, education, transportation and road networks, security, and equal rights and opportunities). Albeit outside the health sector, these are critical considerations adjacent to our findings.

## Conclusion

Reducing maternal deaths is possible in Nigeria, and several approaches would be effective, efficient and cost-effective. Early intensive efforts to improve family planning and control of fertility choices, accompanied by a stepwise effort to scale-up capacity for integrated maternal health services over several years, will save lives and provide equal or greater value than many public health interventions we consider among the most cost-effective (e.g., childhood immunization). With stepwise investments to improve access to pregnancy-related health services and high-quality facility-based intrapartum care, more than 75% of maternal deaths could be prevented. If accomplished over the twelve years, more than 380,000 women could be saved.

## Competing interests

The authors declare that they have no competing interests.

## Authors’ contributions

All authors contributed to the study design, collection and analysis of data, and writing the paper. All authors approved the final version of the manuscript.

## Funding

Authors would also like to acknowledge the John D. and Catherine T. MacArthur Foundation grants (#07-8900-00GSS and #10-97002-000-INP). The funder had no role in the study, design, data collections analysis, decision to publish or preparation of the manuscript.

## Pre-publication history

The pre-publication history for this paper can be accessed here:

http://www.biomedcentral.com/1471-2458/12/786/prepub

## Supplementary Material

Additional file 1 Supplementary materials. Click here for file
