# Supplementary material for: Assessing health and economic outcomes of interventions to reduce pregnancy-related mortality in Nigeria
Source: BMC Public Health. 2012 Sep 14;12:786. doi: 10.1186/1471-2458-12-786 (PMC3491013; doi:10.1186/1471-2458-12-786)
Supplement: Additional file 1 — Supplementary materials. [file 1471-2458-12-786-S1.doc]

Supplemental material

Accompanying the manuscript

Assessing the health and economic outcomes of interventions to reduce pregnancy-related mortality in Nigeria

Daniel O. Erim, MD, MS

Stephen C. Resch MPH, Ph.D

Sue J. Goldie, MD, MPH

| TABLE OF CONTENTS |
| --- |
| Part I. General Background |
| Part II. Analytic Overview |
| Part III. Brief Description of the Global Maternal Mortality Model |
| Part IV. Preliminary Nigeria Modeling Framework |
| Part V: Supplemental results |
| Part VI. References |

**GENERAL BACKGROUND**

Nigeria is the most populous nation in Africa; it has a population of about 151 million individuals , of which about 33 million are women aged 15 – 44 years . Among this group of women, about 55,000 die annually as a result of pregnancy related causes, and this constitutes about 11% of global maternal deaths. With an estimated maternal mortality ratio (MMR) between 800 and 840 maternal deaths per 100,000 live births, these women face a 1 in 23 lifetime risk of maternal death. These estimates closely follow regional averages: sub-Saharan Africa has an estimated maternal mortality ratio of 900 maternal deaths per 100,000 live births and a 1 in 22 lifetime risk of maternal death. Those who survive face a high risk of a pregnancy-related complication such as an obstetric fistula (between 100,000 and one million women in Nigeria are living with untreated fistula.)

Furthermore, several exacerbating factors/events have been identified and include (but are not limited to) the following:

- The sharp variations in maternal health indices that exist across the country’s different geopolitical zones (e.g. MMR averages 165 per 100,000 live births in the Southwest and about 1,500 per 100,000 live births in the Northeast ) and between rural and urban areas (e.g. TFR [national = 5.7, urban = 4.7 and rural = 6.3] and the proportion of women aged 15-19 years who have already begun childbearing [national = 22.9%, urban = 12%, and rural = 28.7%]);
- The low proportion of deliveries supervised by skilled birth attendants (39% in 2008, 34% in 2003 42% in 1999 and 35.6% in 1991 [3]);
- The occurrence of more childbirths outside healthcare facilities (62% in 2008, 66% in 2003, 58% in 1999 and 62% in 1991);
- The Weak Health System characterized by acute shortages in health manpower and poor health infrastructure [2] );
- The poor availability, accessibility and use of contraceptives (met need for family planning = 15% and unmet need = 20%, with reports of occasional scarcity [see **Table 2** and Appendix 2: Selected Recent Nigeria Press]);
- The existence of stringent anti-abortion laws, and attendant high rates of unsafe abortion [10], (see Appendix 2: Selected Recent Nigeria Press) among others.

| **Table 1:** General snapshot Data from The World Bank | |
| --- | --- |
| **Indicator** | **Year 2008** |
| Population, total (millions) | 151.2 |
| Population growth (annual %) | 2.3 |
| Surface area (sq. km) (thousands) | 923.8 |
| Life expectancy at birth, total (years) | 47.9 |
| Mortality rate, infant (per 1,000 live births) | 95.8 |
| Literacy rate, youth female (% of females ages 15-24) | 64.6 |
| GNI (current US$) (billions) | 195.1 |
| GNI per capita, Atlas method (current US$) | 1,170.0 |
| Prevalence of HIV, total (% of population ages 15-49) | 3.1 |
| Percentage of people living below poverty line | 70 |

Although these maternal deaths are largely preventable, many young and apparently healthy Nigerian women die each year. Their death may directly result from bleeding (ante- and postpartum hemorrhage), infection (sepsis), pregnancy induced hypertension (eclampsia and severe preeclampsia), prolonged labor and complications resulting from an unsafe abortion, or indirectly from anemia or malaria.

| **Table 2:** Status and trends | | | | | | |
| --- | --- | --- | --- | --- | --- | --- |
| **Indicators** | **1980** | **1990** | **2000** | **2003** | **2005** | **2008** |
| Maternal mortality ratio (per 100,000) | 516 | 473 | 694 |  | 1,100 [1] | 608 |
| Antenatal care coverage |  |  |  |  |  |  |
| At least 1 visit (%) |  |  | 70.3 | 63.1 |  | 63.7 |
| At least 4 visits (%) |  |  | 47.3 | 47.4 |  | 44.8 |
| Percentage receiving antenatal care from a skilled provider (%) |  | 58.0 | 63.6 | 58 |  | 57.7 |
| Percentage delivered by a skilled provider (%) |  | 32 | 41.6 | 35.2 |  | 38.9 |
| Percentage delivered in a health facility (%) |  | 30.9 | 37.3 | 32.6 |  | 35.0 |
| Adolescent birth rate (%) |  |  |  |  |  |  |
| Unmet need for family planning (%) |  | 20.8 | 13.3 | 26.9 |  | 20.2 |

| **Table 3:** Comparison of Maternal Mortality Ratios Nationally & by Zones in Nigeria | |
| --- | --- |
| **Nigeria (Total)** | **Maternal Mortality (per 100,000 Total Births)** |
| National | 704 |
| Rural | 828 |
| Urban | 351 |
| -Southwest | 165 |
| -Southeast | 286 |
| -South-South | 518 |
| -Northwest | 1,025 |
| -Northeast | 1,549 |
| -North-central | - |

**ANALYTIC OVERVIEW**

The best available data will be synthesized using a computer-based model to assess the costs and health outcomes of different strategies to reduce disability and death due to pregnancy-related complications in Nigeria, while sub-national analyses will be conducted in two geopolitical zones (i.e. South West and Northeast). These zones were selected because the former has the lowest zonal MMR, while the latter has the highest MMR (See **Table 3**). The model captures the natural history of pregnancy and relevant co-morbidities in an individual woman, aggregates clinical outcomes to the population or subgroup level, and reflects setting-specific epidemiology, and access to health care through factors such as infrastructure, human resources, technology, health facilities and transport. We will prioritize data from Nigeria to estimate initial ranges for age-specific probabilities of pregnancy, miscarriage, abortion, risk of maternal complications, and case-specific fatality and morbidity rates. Separate models will be adapted to the Southwest and Northeast geopolitical zones by superimposing data on coverage rates for prenatal care, antenatal care, family planning, facility births and skilled birth attendants (SBAs) in each zone. After integrating assumptions on the availability of transport, facilities, and quality of care, model-projected outcomes (e.g., maternal mortality ratio [MMR], total fertility rate [TFR]) will be compared with available data.

Strategies relied on help in improving coverage of effective interventions and providing access to key services. Interventions could be provided individually, paired, or packaged into a bundle of integrated services; phased approaches involved scaling up access to services over time. Model outcomes include clinical events (e.g., postpartum hemorrhage), aggregate population measures (e.g., life expectancy), and economic costs (e.g., average per person lifetime costs). Monte Carlo simulation was used to track the number of per-woman events such as pregnancies, live births, facility-based births, and maternal complications, allowing estimation of measures and indicators such as TFR, MMR, proportionate mortality ratio (proportion of deaths that are pregnancy-related among women aged 15-45), and lifetime risk of maternal death. We also explored alternative approaches in settings that differ according to underlying maternal risk, health and socioeconomic status, access to health providers, means of referral and transport, and availability of facilities capable of providing different levels of emergency care. Following the reference case recommendations of the Panel on Cost-Effectiveness in Health and Medicine [WHO 2001], as well as guidelines from the DCP2 and WHO [Jamison 2006, WHO CHOICE], strategies are first ranked in terms of increasing costs and benefits. Strategies are considered “inefficient” or dominated if they were more costly and less effective, or more costly and less cost-effective, than an alternative strategy. Strategies that cost less than the status quo are considered ‘cost saving’. Incremental cost-effectiveness ratios are calculated for *all other* strategies (those that are not cost saving and those that are not dominated). The incremental cost-effectiveness ratio is defined as the additional cost of a specific intervention(s) divided by its additional clinical benefit compared with the next least expensive strategy. While costs and benefits in the model are discounted 3% annually, sensitivity analyses were conducted to assess the impact of parameter uncertainty on our results.

**THE MODEL**

The computer-based Global Maternal Health Policy Model simulates the natural history of pregnancy (both planned and unplanned) as well as pregnancy- and childbirth-associated complications. This model defines health states to reflect important characteristics that affect prognosis, quality of life, and resource use. The time horizon incorporates a woman’s entire lifetime and is divided into equal time increments during which women transition from one health state to another. Non-pregnant girls enter the model and in each time period may become pregnant depending on age, use of contraception, and clinical history. Once pregnant, women have a chance of spontaneous abortion (i.e. miscarriage), induced abortion, or continued pregnancy. A proportion of induced abortions will be unsafe (i.e. conducted by untrained personnel or in an unsafe environment). Labor and delivery may be associated with a direct complication of pregnancy (e.g., hypertensive disorders of pregnancy, obstructed labor, hemorrhage, sepsis), while case fatality rates are conditional on the type and severity of complication (e.g., moderate sepsis requiring antibiotics in bEmOC versus severe hemorrhage requiring blood transfusion in cEmOC) and underlying comorbidity. Nonfatal complications include neurological sequelae, rectovaginal fistula, severe anemia, and infertility. In addition to death from maternal complications, women face an annual risk of death from age-specific all-cause mortality.

Strategies in the model to reduce maternal mortality consist of improving coverage of effective interventions, which may be provided individually or packaged as integrated services. In addition to family planning, antenatal care, and safe abortion, the model includes both intrapartum interventions that reduce the incidence of a complication (e.g., Misoprostol for postpartum hemorrhage [PPH]) and those that reduce the case fatality rate through appropriate management in a referral facility.

The effectiveness of interventions depends, in part, on access to specific services (e.g., trained skilled birth attendant [SBA]) and to specific levels of facilities (e.g., comprehensive emergency obstetrical care [cEmOC] with capacity for blood transfusion). Accordingly, the ultimate impact of interventions depends on several setting-specific factors. These include the delivery site, presence of birth attendant(s), quality and type of referral facility, as well as successful referral when necessary. The model therefore explicitly considers the location of delivery, type of assistance, access to basic/comprehensive obstetric care, and the ability to overcome a series of barriers around the timing of delivery (e.g., recognition of referral need, reliable transport, timely treatment at an appropriate facility); these factors collectively determine the health services a woman can access and the specific interventions that would be included.

Delivery setting is differentiated by provider (e.g., family member, traditional birth attendant [TBA], or SBA) and by site (e.g., home versus facility). Facility levels are categorized as follows: birthing centers or health centers, which cannot provide all services necessary to qualify as a basic emergency obstetrical care (bEmOC) facility, but are staffed with SBA who provide expectant management of labor and more reliable referral when necessary than with delivery at home; facilities with bEmOC capacity (e.g., first referral units); and facilities with cEmOC capacity (e.g., district hospitals).

| 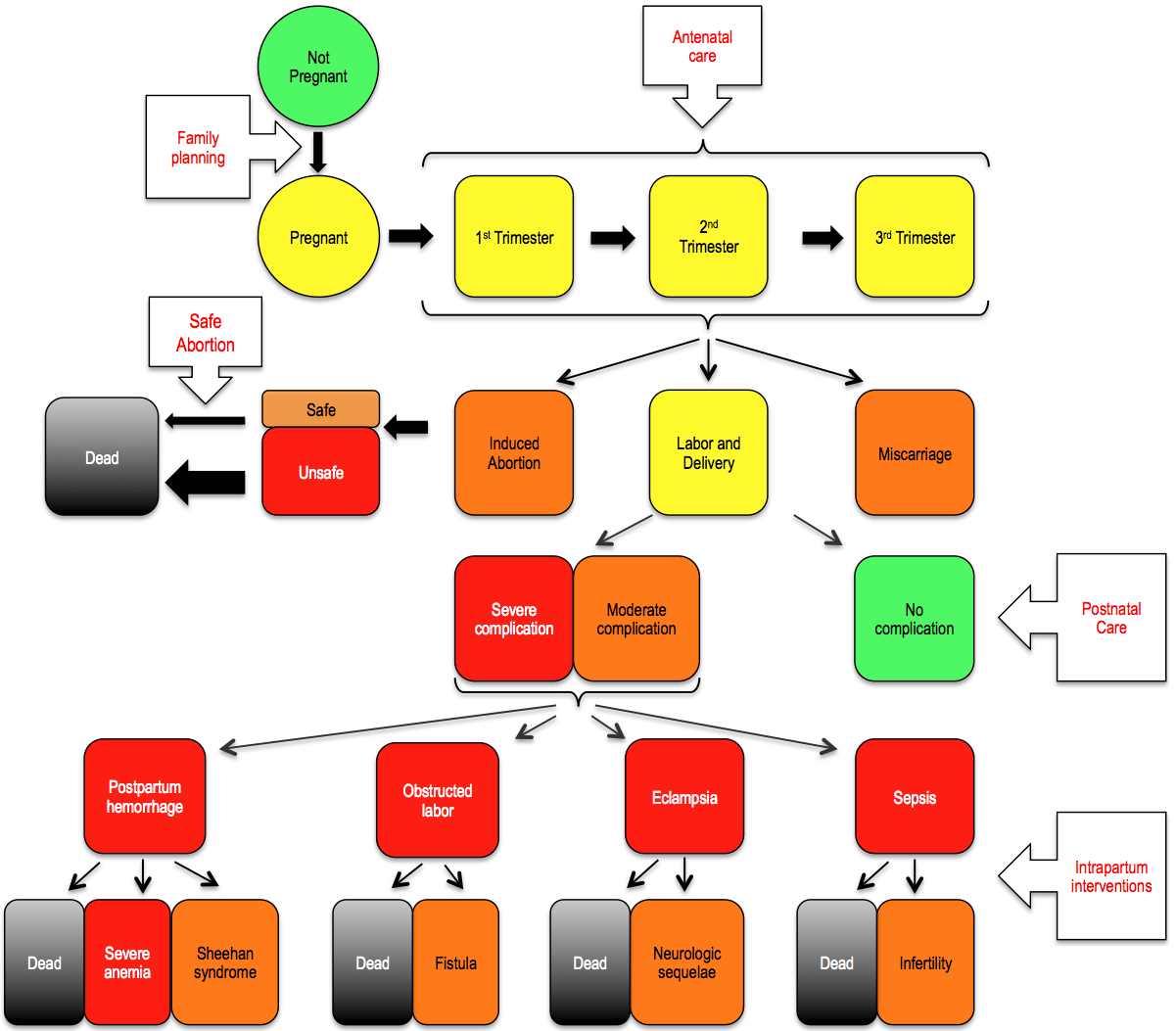 |
| --- |
| Figure 1: Schematic of the model. The model simulates the natural history of pregnancy and related events/complications. General interventions (red font) include family, antenatal (with treatment of anemia), safe abortion, intrapartum care (e.g., active management of labor in basic and comprehensive EmOC facilities) and postpartum care. While case fatality rates of complications depend on severity and comorbidity, interventions reduce the incidence, severity and or case fatality rate of a complication through appropriate treatment. |

Facilities capable of bEmOC are assumed to be capable of administering injectable antibiotics, oxytocics, sedatives and anticonvulsants as well as performing manual removal of placenta/retained products, and assisted vaginal delivery. Facilities capable of cEmOC are able to provide bEmOC services as well as blood transfusion, Cesarean deliveries, and management of advanced shock. This model also allows us to evaluate phased approaches that involve scaling up access to services over time, of which we designate such stepwise investments in infrastructure as “upgrades”. In addition to reducing the prevalence of unsafe abortion and unmet need for family planning, these strategies incrementally shift home births to facilities, increase skilled attendants, and improve access to, and quality of, emergency obstetric care. For women delivering at home or in birthing centers, these strategies also improve recognition of the need for referral, access to transport, and expedient referral to an appropriate facility. The facility categories are flexibly modeled such that distinctiveness of the public health infrastructure in different settings (country, state, rural versus urban areas) can be accurately represented in terms capacity and cost. Figure 3 is a stylized example of how public health facilities can be superimposed on our general model framework.

Facilities can also be grouped as follows: primary-level facilities, which may not have all bEmOC functions but could serve as birthing centers with SBA, 24-hour intrapartum care and a reliable referral system (e.g. health centers, primary health centers [PHC], maternity homes etc.; secondary facilities, which have bEmOC capacity (e.g., first referral unit [FRU], community health center [CHC], some General Hospitals and private clinics) and some may provide cEmOC services; tertiary facilities, which have cEmOC capacity (e.g., Teaching Hospitals, Federal Medical Centers, private Specialist Hospitals, some General Hospitals). Facilities can also be hybrid.

We recognize that some tertiary sites do not have blood banks, that some secondary sites may provide C-section and that some primary-level facilities do not offer 24-hour care. We also recognize that for strategies that include stepwise investments in infrastructure/facility improvement, not all facilities will be fully upgraded. However, because the costs, functions and staffing are fairly closely aligned with basic or comprehensive EmOC capacity, this simple categorization captured the most important dimensions for the purpose of this analysis.

Models were programmed using TreeAge Pro 2008 (*TreeAge Software Inc., Williamstown MA*) while analysis was done using Microsoft Excel 2007 and Visual Basic for Applications 6.5 (*Microsoft Corp., Redmond WA*) on IBM/Lenovo Dual-Core VT Pro Desktop computers running Microsoft Windows XP. We used Monte Carlo simulation to generate the number of per woman events such as pregnancies, live births, facility-based births, and maternal complications. This output is useful for bothcalibration exercises, as well as assessing internal consistency and projective validity of the model. We used second order Monte Carlo simulation to assess parameter uncertainty.

| **Table 4:**  Framework to differentiate facilities in Nigeria (from the National Primary Health Care Development Agency of Nigeria). FCT = Federal Capital Territory also known as Abuja; LGA = Local Government Area). | | | | |
| --- | --- | --- | --- | --- |
| **Health facilities** | **Level** | **Description** | **Levels of management** | **Expected numbers** |
| Teaching hospitals  Federal Medical Centers | 3rd level (Tertiary) Highest level | There is at least one in each state of the Federation. They have specialists and residents in most specialties including Ob/Gyn. Government owned (Federal). | Federal Government | 1 per State. Therefore in 36 States + FCT, 37 |
| Specialist hospitals | 3^rd^ level Specialists | They have fewer specialists and may have a/several resident doctor(s)/medical officer(s). | State government or private sector |  |
| General hospitals Comprehensive Health Centers | 2^nd^ level (Secondary) | Manned by medical officers. May have some specialists. Government-owned (State). | State Government | 1 per LGA. Therefore a minimum of 774 will be expected |
| Private clinics | 2^nd^ level (Secondary) | Privately owned medical facilities manned by 1 or more medical officers. | Private sector |  |
| Primary Health care centers | 1st level (Primary) | Government owned health facilities with nurses, midwives and community health worker. | Local Government | 1 per ward with average of 10 wards per LGA, a total of 7,740 will be expected |
| Private clinics | 1^st^ level (Primary) | Privately owned health facilities with nurses, midwives and community health worker. | Private sector |  |
| Health clinics | 1^st^ level (Primary) | Government owned health facilities with community health worker. They may have few nurses and/or midwives | Local government and District/Ward Development Committee | 1 per group of villages/ neighborhoods with about 1,500 – 2000 persons |
| Health posts | 1^st^ level (Primary) | Government owned health facilities with one or more community health workers | Village/Community Development Committee | 1 per village or neighborhood of about 200 – 500 persons. As many as the number of villages |

IV. Preliminary Nigeria Modeling Framework

| **Subsection A** |
| --- |
| **Data and Assumptions:**  Initial Natural History Parameters**:** The best available data are sought on clinical parameters governing the natural history of pregnancy. Examples of required model inputs include the age-specific probability of pregnancy, miscarriage, unsafe and safe abortion; incidence, morbidity, and case fatality rates for each maternal complication (PPH, sepsis, obstructed labor, hypertensive disorders of pregnancy); prevalence of co-morbidities (e.g. anemia).  Intervention Effectiveness: The effectiveness of interventions to reduce the incidence of complications and/or reduce case fatality rates is from published data, and varies by complication type and severity. Initial estimates assume an intervention can be delivered appropriately, but are then modified according to several setting-specific factors. These include delivery site, presence of birth attendant, quality and type of referral facility, as well as successful referral when necessary |
| **Subsection B** |
| **Data:**  Coverage rates and Selected Services: Data on coverage rates for interventions, facility births, skilled birth attendants, antenatal care, and family planning for the country and subnational analysis for 2 selected geopolitical zones for model validation.  **Data and Assumptions:**  Barriers to Effective Referral: Effective referral relies on the ability to overcome three critical delays  (a) recognition of need for referral and willingness to be referred (by provider and delivery location);  (b) expedient transfer to referral facility (determined by distance, affordability, available transport);  (c) timely treatment in an appropriate facility capable of high-quality emergency obstetrical care (e.g., 6 signal functions in bEmOC, blood transfusion and surgery in cEmOC). |
| **Subsection C** |
| **Calibration: Exercises**:  Calibration targets include the distribution of causes of maternal mortality (e.g., PPH, obstructed labor, sepsis), maternal mortality ratio (MMR), total fertility rate (TFR) etc. After integrating assumptions about the availability of health services, model-projected estimates of MMR, TFR, and distribution of direct causes of maternal mortality are compared to empiric data. Selected uncertain parameters (such as the case fatality rates conditional on severity of complication) are varied across a pre-specified plausible range, in a systematic fashion, to ensure that the output is consistent with key empiric indicators.  **Model Performance:**  Model performance is assessed by comparison of model-based projections with independent measures such as life expectancy, proportionate mortality ratio, and population-based outcomes. Projective validity of the empirically-calibrated model is further assessed by simulating two geopolitical zones (i.e. Southwest and Northeast), and comparing projected maternal health indicators with reported data |
| **Subsection D** |
| **Sources of Non-Literature-based Information (e.g., surveys):**  Preliminary running list of sources. |

**SUBSECTION A**

**Data and Assumptions: Age-specific probability of pregnancy**

First, we modeled the natural history of pregnancy, and then we inserted setting-specific parameters such as fertility rates, age of onset of sexual activity and contraceptive use (by age and method). In order to estimate the fertility rate in the absence of any family planning, we used data from places where contraceptive use is low, maternal mortality ratio (MMR) is high (~1,600 deaths per 100,000 live births), and access to modern health care is limited (< 5%). We used data on family planning, abortion, and demographics (e.g. Afghanistan’s crude birth rate of 308 pregnancies per 1,000 population) to approximate an average annual natural fertility rate of 31% . The model also allows for insertion of age-specific data for fertility and use of contraception. We however made several assumptions which include the following: that 15% of all pregnancies end in spontaneous abortion, of which approximately one-third result in incomplete abortion requiring medical intervention ; that women with long-term complications such as infertility or untreated obstetric fistula do not become pregnant again and that women with complications that were treated (e.g., severe anemia, surgically repaired fistula) could still become pregnant.

**Anemia**

Among women of reproductive age in Nigeria, the overall prevalence of anemia in pregnancy has been estimated to be 60% , down from 66.7% estimated by the WHO in 1993 (see **Table 7**) . When compared to women without anemia, the relative risk of death from maternal complications is 3.5 times greater in women with severe anemia and 1.35 times greater in women with moderate anemia, and antenatal care presents an opportunity to detect and treat anemia. We assumed severe and moderate anemia were associated with higher risks of death from pregnancy- and delivery-related complications. Although anemia differentially affects mortality from postpartum hemorrhage, sepsis, and complications following unsafe abortion, we conservatively assumed that severe anemia did not impact the case fatality rate of untreated obstructed labor.

| **Table 5:** Anemia prevalence | |
| --- | --- |
| **Women in Nigeria** | **Women (aged 15-49) who are anemic (%)** |
| -All women | 62 |
| -Pregnant Women | 60 |
| **Pregnant women by zone:** |  |
| -Southwest | 33 – 77 |
| -South-south | 56 |
| -Southeast | 40 |
| -North-central | - |
| -Northwest | 48 |
| -Northeast | 52 - 72 |

**Pregnancy-related complications:**

**Incidence, case fatality rates and interventions to reduce incidence and mortality**

Initial estimates of incidence and case fatality rates of pregnancy-related complications were obtained from published data, and plausible ranges for sensitivity analysis were derived from systematic review of the literature. Adjustments to case fatality rates were based on severity of complications and underlying anemia, and the effectiveness of interventions to reduce the incidence of complications and case fatality rates were derived from published studies. The effectiveness depends, in part, on access to specific services (e.g. SBA) and/or to specific facilities (e.g. cEmOC capacity for blood transfusion).

The ultimate impact of interventions therefore depends on several setting-specific factors. These include the site of delivery, presence of a skilled birth attendant, quality and type of referral facility and successful referral when necessary. Data on facility births, skilled birth attendants, family planning and antenatal care were from country-specific surveys. A summary table is provided below, and the sections that follow provide information on the source and rationale for the baseline initial estimates, the adjusted estimates and the range for sensitivity analyses.

| **Table 6:** Overview of Parameters and Ranges Evaluated in Sensitivity Analysis | | | | | | |
| --- | --- | --- | --- | --- | --- | --- |
| **Complications** | | **Hemorrhage** | **Obstructed Labor** | **Hypertensive disorders** | **Sepsis** | **Unsafe abortion** |
| Incidence | | 0.114 | 0.047 | 0.035 | 0.050 | 0.128 |
|  | *Range* | 0.051 – 0.228 | 0.030 – 0.074 | 0.025 – 0.050 | 0.043 – 0.060 | 0.050 – 0.250 |
| CFR * (initial) | | 0.010 | 0.007 | 0.017 | 0.013 | 0.003 |
|  | *Range* | 0.007 – 0.013 | 0.005 – 0.009 | 0.012 – 0.022 | 0.009 – 0.017 | 0.002 – 0.004 |
| CFR* (adjusted) | | **0.023** | **0.019** | **0.021** | **0.028** | **0.009** |
|  | *Range* | 0.016 – 0.030 | 0.013 – 0.025 | 0.015 – 0.027 | 0.020 – 0.036 | 0.006 – 0.012 |
| *Range used* | | 0.007 – 0.030 | 0.005 – 0.025 | 0.012 - 0.027 | 0.009 – 0.036 | 0.002 – 0.012 |
| **Effectiveness (prevention, management and treatment)** | | | | | | |
| **↓** Incidence | | 50%, 75% | ---- | ---- | 25%, 50% | ---- |
|  | *Range* | 25% - 91% | ---- | 25%-50% | 0% - 60% | 0% - 100% |
| **↓** CFR | | 75% | 95% | 59% | 90% | 98% |
|  | *Range* | 60% - 90% | 76% - 100% | 45% - 95% | 63% - 93% | 50% - 100% |

**Adjusting case fatality rates for heterogeneity in severity and co-morbidity**

Baseline estimates for cause-specific case fatality rates were from the Disease Control Priorities Project (DCP2) and are thus: maternal hemorrhage (1%); sepsis (1.3% ); hypertensive disorders of pregnancy (1.7%), obstructed labor (0.7%). These case fatality rates are lower than those reported in some studies, implying that they could be underestimates . We assumed that some of the variations reported in the literature are due to the heterogeneity in severity as well as use of estimates from studies with small sample sizes. Case fatality rates were thus adjusted based on the severity of complication and underlying anemia. We assumed that life-threatening complications (i.e. those requiring cEmOC-level services) were associated with a higher case fatality rate (in the absence of treatment) than non-life-threatening complications. This relative risk was determined through a number of calibration exercises. The model was first parameterized using the best natural history data available, and then adjusted to reflect the current standard of care in Nigeria. Thereafter, the relative risk was allowed to vary such that the model fit multiple epidemiologic targets simultaneously, including the MMR, life expectancy, total fertility rate, and distribution of maternal mortality causes. In addition, we assumed that underlying severe anemia increased the relative risk of mortality from severe hemorrhage, sepsis and abortion; the increase ranged from 1.5 to 3.0 . Assumptions for the proportion requiring bEmOC and cEmOC are described below. Among the estimated 15% of pregnant women in developing countries who experience pregnancy-related complications, 7% require care at centers with surgical capacity (cEmOC) and 2-3% will require surgery . Initial estimates of what proportion of complications require basic versus comprehensive EmOC care were derived from a WHO study.

| **Table 7: Proportion of complications in pregnant women that require bEmOC and cEmOC care.** | | |
| --- | --- | --- |
| **Johns (2007)** | **Require bEmOC** | **Require cEmOC** |
| Hypertensive disorders | 85.8% | 14.2% |
| Obstructed labor | 8% (assisted delivery) | 92% (cesarean section) |
| Postpartum hemorrhage | 72.5% | 27.5% (25% transfuse, 2% surgery) |
| Puerperal sepsis | 63% | 10% transfusion; 27% shock |

In setting-specific models (e.g. Southwest versus Northeast geopolitical zones) these proportions may be altered either by using data on the availability and distribution of EmOC facilities and extent of training of their personnel, or from insights gained during the calibration exercises (Subsections B and C). In certain cases, we have published data to assist with modifying estimates. For example, Johns et al assumed that 85.8% of Eclampsia cases (HTN) require bEmOC, while 14.2% require cEmOC . This estimate is similar to an earlier study, which showed that patients with severe Pre-eclampsia and eclampsia required treatment with intravenous hydralazine and magnesium sulfate, and that approximately 10% of all cases were assumed to require emergency cesarean section . Initial proportions (and ranges) used in Nigeria are provided below (**Table 10**). Sensitivity analyses were conducted to assess the implications of using the upper and lower bounds.

| **Table 8:** Assumptions about complications requiring bEmOC and cEmOC | | |
| --- | --- | --- |
| **Complication** | **Require bEmOC** | **Require cEmOC** |
| Hypertensive | 85.8% (75 - 90%) | 14.2% (10 - 25%) |
| Obstructed labor | 31.0% (5 - 35%) | 69% (65 - 95%) |
| Postpartum hemorrhage | 65.6% (60 - 72.5%) | 34.4% (27.5 - 38%) |
| Puerperal sepsis | 63.0% (60 - 75%) | 37% (25 - 40%) |

Using the assumptions above, we collated literature-based case fatality rates and adjusted these rates based on severity (e.g., necessity of cEmOC, need for transfusion, delay in reaching care, underlying moderate anemia, underlying severe anemia etc.) For example, to calculate implied average CFR for PPH, we did the following; we took the baseline CFR of 1%, applied relative risks based on severity and then weighted these based on the percentage of women who would face that risk.

| **Table 9:** Adjusted CFR and Expanded Range for Sensitivity Analysis* (in the absence of interventions) | | | | | |
| --- | --- | --- | --- | --- | --- |
|  | **Hemorrhage** | **Obstructed Labor** | **Hypertensive disorders** | **Sepsis** | **Unsafe Abortion** |
| CFR * (initial) | 0.010 | 0.007 | 0.017 | 0.013 | 0.003 |
| *Range* | 0.007 – 0.013 | 0.005 – 0.009 | 0.012 – 0.022 | 0.009 – 0.017 | 0.002 – 0.004 |
| CFR* (adjusted) | **0.023** | **0.019** | **0.021** | **0.028** | **0.009** |
| *Range* | 0.016 – 0.030 | 0.013 – 0.025 | 0.015 – 0.027 | 0.020 – 0.036 | 0.006 – 0.012 |
| ***Range used**** | **0.007 – 0.030** | **0.005 – 0.025** | **0.012 - 0.027** | **0.009 – 0.036** | **0.002 – 0.012** |

**Hemorrhage**

**Incidence and case fatality rate**

In a systematic review of 34 datasets representing over 35,000 maternal deaths, Khan et al. found hemorrhage to be the leading cause of death throughout the world, responsible for the highest proportion of deaths in Asia and Africa and accounting for 1.4% to 49.6% of all maternal deaths. In Nigeria, hemorrhage is the leading cause of maternal mortality and contributes up to 23% of all maternal deaths . Maternal hemorrhage is categorized according to its timing in relation to delivery: antepartum, intrapartum, or postpartum. The etiologies and management of maternal hemorrhage differ among these three categories.

Initial estimates for the overall incidence of PPH were based on data from the WHO Global Burden of Disease study, and the risk of PPH was modified to reflect the study’s assumptions. The incidence of PPH (defined as blood loss of >1000ml) in the oxytocin arm was 2.85% within 1 hour postpartum in women who were actively managed, as estimated from the MISO trial. We assumed that the incidence of PPH in women who are managed expectantly by a skilled birth attendant would be twice as high as found in the MISO trial, or 5.7% of births , and the incidence in women who gave birth without skilled attendance would be twice as high as those with skilled birth attendance.

Extrapolating from these data, we made the following assumptions:

1. all births in a facility with emergency obstetrical care would be actively managed, and had a 2.85% risk of PPH
2. all other births with a skilled attendant would be expectantly managed, and had a 5.7% risk of PPH, and
3. all births attended by a family member, a traditional birth attendant or no one, would be associated with an 11.4% risk of PPH (approximating the reported range of 8 - 15% in the literature).

The initial estimate for the case fatality rate (CFR) was from a review conducted by the Disease Control Priorities Project (DCP2) which reported an average CFR of 1%. This estimate was adjusted for case severity and underlying morbidity (e.g., severe anemia) while calibrating the model to fit multiple epidemiologic targets simultaneously. [See section above on “*Adjustment of case fatality rates for heterogeneity in severity and co-morbidity*”]. Adjusting our CFR widened its plausible range. While the literature-based CFR range was 0.007 – 0.013 (based on 0.01 or 1% average CFR from DCP2), the expanded plausible range was 0.007 – 0.030 (based on our adjustment of a CFR of 0.023 [2.2%]). Model-projected mortality due to maternal hemorrhage, as well as MMR, TFR, and calendar deaths for 2008, closely approximated the empiric data.

To account for the uncertainty in our initial estimates, we established a plausible range for all the above parameters based on our literature review. There have been several studies on the incidence and case fatality rate of maternal hemorrhage. A Cochrane review demonstrated that when compared to expectant management, active management with oxytocin results in a relative risk of 0.33 for PPH within the first 24 hours. This review has since been withdrawn due to concerns regarding the validity of these findings. An updated analysis is currently underway to ensure the use of more recent data. A Cochrane review comparing oxytocin to no uterotonics found a 39% reduction in incidence of PPH (RR 0.61). When active management was used in both trial arms, the same review found a relative risk of 0.33, and when expectant management was common to both trial arms, a relative risk of 0.73. A study published in 2002 comparing active management with expectant management found a relative risk of 0.8 for blood loss > 500ml. While severe PPH was not an endpoint captured in this trial, we would expect a slightly lower RR for blood loss > 1000ml. A more recent randomized control trial of a small number of women who gave birth at a maternity unit in Iran compared active management with oxytocin to expectant management and found conflicting evidence regarding the optimal method by which to manage the third stage of labor. This trial found that active management did not decrease blood loss during the third stage of labor but did decrease the duration of this stage. Active management was associated with increased blood loss during the fourth stage of labor. However, severe PPH was not an endpoint captured within this analysis.

In a systemic review of randomized trials, the prophylactic administration of oxytocin reduced the risk of severe PPH from 7% to 4.3% (RR 0.61), and the relative risk of severe PPH when using Misoprostol compared to placebo (when an outlier study was excluded) was 0.77 for 600 mg and 0.54 for 400 mg. There was wide variation in the effect of Misoprostol but all studies showed some effectiveness compared to placebo. A systemic review covering the period 1997-2006 included 24 studies from the WHO database covering a period of 1997-2002 and an additional 166 reports assessed (from 2003-2006) with 14 included (total = 38) for a total of 224 datasets. These were stratified to those that reported PPH (n=120) and those that reported severe PPH (n=70), with severe PPH being defined as blood loss >1,000 ml. Overall quality was deemed adequate for ~47% of PPH datasets and ~59% of SPPH datasets. Overall prevalence of PPH was 6.09% (CI 6.06 to 6.11), with 10.55% when the blood loss was measured objectively. Overall prevalence of severe PPH was 1.86% (CI 1.82 to 1.90), with 3.04% when the blood loss was measured objectively. A high degree of heterogeneity was reported even in subgroups with similar characteristics. Severe PPH was reported at 3.84% for expectant management alone (CI 3.31-4.37) in 6 datasets of 4,999 women, 2.99% (2.80-3.18) for active management alone in 21 datasets with 30,608 women, 2.47% (2.06-2.88) for uterotonic before delivery in 11 datasets with 5,585 women, and 2.08% (1.39-2.77) for uterotonic after delivery in 2 datasets with 1,635 women. (See table)

**Reduction in mortality**

The incidence of maternal hemorrhage is dependent on the delivery setting, the use of expectant or active management, and the use of Misoprostol. The model represents a range of approaches to *reduce mortality* from PPH: first, on the basis of delivery setting and use of expectant or active management of labor; second, by successful referral and access to quality care in an appropriate facility with basic or comprehensive emergency obstetric care; third, by the use of temporizing measures such as anti-shock garment to reduce blood loss en route to an adequate facility; fourth, by the use of Misoprostol after PPH at home or in a birthing center, to reduce total blood loss; fifth, by the use of Misoprostol in the community setting, at home or in a birthing center or sub-center, to prevent PPH. Data for the first, second and third options are far more readily available than the fourth and fifth options, which informed our choice to focus on the first three for the present analysis. We include one exploratory analysis of community-based SBA-administered Misoprostol. We assumed optimal treatment of maternal hemorrhage in an appropriate facility with EmOC capacity consisted of intramuscular or intravenous oxytocin immediately after delivery, uterine massage, repair of any perineal or vaginal tears, and fluid replacement or blood transfusion. Consistent with assumptions made by Adam et al. and Graham et al. we assumed an average reduction of 75% in the CFR . We varied this estimate from 60% to 90% for optimal management (i.e., bEmOC or cEmOC as necessary for severity).

| Table 10: Studies on maternal hemorrhage that contribute to plausible range for calibration exercises and sensitivity analyses | | | | | |
| --- | --- | --- | --- | --- | --- |
| **Strategies to reduce incidence of maternal hemorrhage** | Relative Risk (RR) | | % Reduction in Risk | | |
|  | RR | 95% CI | % | Lower | Upper |
| **Active management of labor** | | | | bound | bound |
| Active management with oxytocin vs. expectant management for incidence of severe PPH in 1st 24 hours | 0.50 |  | 50% |  |  |
| Active vs. expectant management, severe PPH | 0.34 | (0.14, 0.87) | 66% | 13% | 86% |
| Active vs. expectant management, primary maternal blood loss> 500ml | 0.34 | (0.27, 0.44) | 66% | 56% | 73% |
| Active vs. expectant management, secondary maternal blood loss> 500ml | 0.33 | (0.13, 0.84) | 67% | 16% | 87% |
| Active management & misoprostol by SBA in HC | Lower blood loss vs. control, but few PPH (0.7% vs. 0.8%) | | | | |
| Active vs. expectant management, severe PPH |  |  | 50% |  |  |
| Oxytocin vs. no uterotonics, severe PPH, with active management for both | 0.33 | (0.14, 0.77) | 67% | 23% | 86% |
| Active vs. expectant management, PPH (>=500ml) only | 0.8 | (0.7, 0.9) |  |  |  |
| Misoprostol vs. oxytocin w/ active management, severe PPH | 1.39 | (1.19, 1.69) | -39% | -69% | -19% |
| Active vs. expectant management, no endpoints on severe PPH | Active management – while no decrease in blood loss, there was a decrease in the duration of 3rd stage and an increase in blood loss in 4th stage | | | | |
| Active management and risk of PPH | No effectiveness data | | | | |
| Expectant management w/ SBA vs. nothing, severe PPH |  |  | 50% |  |  |
| Oxytocin vs. no uterotonics, severe PPH, with expectant management for both | 0.73 | (0.49, 1.07) | 27% | -7% | 51% |
| **Oxytocin** | | | | | |
| Oxytocin vs. no uterotonics, severe PPH | 0.61 | (0.44, 0.87) | 39% | 13% | 56% |
| Oxytocin vs. no uterotonics, severe PPH, RCT only | 0.72 | (0.49, 1.05) | 28% | -5% | 51% |
| Oxytocin vs. no uterotonics, outcome of severe PPH (>=1000ml blood loss) | 0.57 | (0.41, 0.79) | 43% | 21% | 59% |
| Oxytocin vs. no uterotonics, severe PPH |  |  | 61% |  |  |

**Misoprostol**

Among most of the studies that have assessed misoprostol, there are variations in methods and clinical practice (e.g., measurement of blood loss, management of the third stage of labor, and use of uterotonics to prevent versus manage PPH). These variations make direct comparison difficult.  Gulmezoglu et al. reported oxytocin to be more effective than misoprostol in reducing acute and severe PPH. However, the study included several developed countries, and restriction of analysis to data from developing countries showed that the risks are similar for oxytocin and misoprostol. More recent studies shown the benefits of misoprostol in reducing the risk of PPH. Derman et al. reported the results of a placebo-controlled trial where auxiliary nurses/midwives at home or in village birthing centers administered Misoprostol (600 mcg) or a placebo orally, in the context of expectant management. They found a statistically significant reduction in risk for acute PPH, severe PPH, use of additional uterotonics, transfer to higher-level facility and transfusion in the Misoprostol group versus the placebo group. Another large study (n > 600) by Hoj et al. in Guinea-Bissau showed a reduction in the risk for severe PPH although not acute PPH.  The incidence of acute PPH (blood loss > 500) was high for both Misoprostol and placebo groups (45% and 51%) as was the incidence of severe PPH (11% and 17%).  Walraven et al. reported results of a large study in the Gambia (n> 1000) comparing TBA-administered Misoprostol (600 mcg) to ergometrine (2 mg). Both drugs had similar risks of acute PPH (11-12%) and severe PPH (0.3-0.7%), but lower blood loss compared to placebo. Another large study (n>1000) by Chandhiok et al. compared the effectiveness of Misoprostol (600 mcg administered orally) to methergine (administered orally and intramuscularly). The risk of acute PPH were found to be very low and similar in both groups (0.70-0.8%).

We also found that prophylactically administering Misoprostol (600mcg) was more effective than placebo at preventing PPH in community births (RR = 0.59), but not in hospitals (RR = 1.23). This was reinforced by a Cochrane Review, which concluded that although Misoprostol was less effective than oxytocin and associated with higher rates of shivering and fever, it showed promising results when compared to the placebo. A review of the evidence supporting guidelines showed that for prevention of PPH, active management reduced risks during the third stage of labor, and that Misoprostol should only be used if oxytocin is unavailable. From a systematic review of randomized trials, Hofmeyr and Gulmezoglu found that there was no difference in severe morbidity between Misoprostol and other conventional uterotonics, although those who were given Misoprostol did experience more side effects. However, on exclusion of an outlier study, results showed less blood loss with Misoprostol than with the placebo provided during the trial. It also showed that prophylactic administration of oxytocin reduced the risk of severe PPH from 7% to 4.3% (RR 0.61), and the relative risk of severe PPH when using Misoprostol compared to placebo was 0.77 for 600 mg and 0.54 for 400 mg. This review did not answer the question of whether the relative mortality reduction owing to Misoprostol preventing PPH-related deaths was offset by an increase in mortality caused by the drug. In addition, the review did not compare active versus expectant management.

| **Table 11:** Effectiveness of Misoprostol | | | | | |
| --- | --- | --- | --- | --- | --- |
|  | Relative Risk (RR) | | % Reduction in Risk | | |
| **Intervention** | RR | 95% CI | % | Lower | Upper |
| Misoprostol vs. no uterotonic; severe PPH |  |  | 77% |  |  |
| Misoprostol vs. placebo; severe PPH | 0.63 | (0.44, 0.91) | 37% | 9% | 56% |
| Misoprostol vs. Injectable uterotonics; severe PPH | 1.34 | (1.16, 1.55) | -34% | -55% | -16% |
| Misoprostol vs. placebo in community births; severe PPH | 0.59 | (0.41, 0.84) | 41% | 16% | 59% |
| Misoprostol vs. placebo in hospital births; severe PPH | 1.23 | (0.86, 1.74) | -23% | -74% | 14% |
| Misoprostol vs. placebo/no treatment; severe PPH | Heterogeneity - lack conclusive evidence | | | | |
| Misoprostol vs. placebo/no treatment (early trials); severe PPH | Early trials show no reduction | | | | |
| Misoprostol vs. placebo/no treatment (later trials); severe PPH | 0.31 | (0.1, 0.94) |  | | |
| Misoprostol vs. injectable uterotonics; severe PPH | 1.32 | (1.16, 1.51) |  |  |  |
| RCT: Misoprostol with SBA vs. placebo; severe PPH 2 hrs | 0.20 | (0.04, 0.91) | 80% | 9% | 96% |
| RCT: Misoprostol with SBA vs. placebo; PPH (≥ 500ml) 2 hrs | 0.53 | (0.39, 0.74) | 47% | 26% | 61% |
| Misoprostol vs. placebo; severe PPH | 0.66 | (0.45, 0.98) | 34% | 2% | 55% |
| Misoprostol vs. placebo; severe PPH | 0.48 | (0.09, 2.59) | 52% | -159% | 91% |

**Inclusion of antepartum hemorrhage**

Johns et al (2007) estimate that 2.2% of pregnancies will be complicated by antepartum hemorrhage requiring management: that 0.11% of pregnancies will be complicated by antepartum hemorrhage requiring caesarean section and that 0.726% of pregnancies will be complicated by antepartum hemorrhage requiring transfusion. As with postpartum hemorrhage, the onset and course of antepartum hemorrhage can be unpredictable: a recurrent bleed can occur at any time and to any severity. Antepartum hemorrhage was not considered as a *separate category* in our model because we felt there was insufficient data on its epidemiology, natural history, and the impact of interventions *in developing countries*. [For these same reasons, estimates of death and disability attributable to antepartum hemorrhage were not included in the WHO’s global burden of maternal hemorrhage]. However, we *did* calibrate to observed data on the distribution of deaths by cause in Nigeria [see Subsection C.]

In contrast to postpartum hemorrhage, the frequency of antepartum hemorrhage has been difficult to establish at the population level in developing countries due to a lack of widely accepted diagnostic criteria for this condition and reliable ascertainment, which is grossly affected by the quality and availability of maternal care. Similarly, empirical data regarding the natural history of antepartum hemorrhage are also lacking as shown by the following examples:

1. The proportion of antepartum hemorrhages that present as severe or life threatening is unknown
2. The proportion of cases that ultimately require transfusion and/or cesarean section is unknown.
3. The percentage of cases that resolve only to recur later on is unknown.
4. The mortality or morbidity risk in the absence of medical care is unknown
5. Data on the impact of interventions targeting antepartum hemorrhage is scarce.

In developed countries, management of antepartum hemorrhage is frequently determined on a case-by-case basis since its etiology varies and management is dependent on multiple factors including etiology, the status of the mother and fetus, the amount of bleeding, gestational age, and, in the case of placenta previa and abruption, the degree of separation between the uterus and the placenta. In developed countries, where comprehensive maternal care is not only high quality but also widely and promptly available, the mortality risk of antepartum hemorrhage has been reduced to <1%. This low mortality risk is attributable to a highly vigilant approach to this condition, generally consisting of the following:

1. Emergency caesarean section for patients with refractory hemorrhage, poor fetal status, or significant bleeding after 34 weeks gestation;
2. Hospitalization with close monitoring and supportive care for actively bleeding patients;
3. Expectant management as an inpatient (or outpatient if the patient lives within 5-10 minutes of a comprehensive medical center) with close follow-up and planned caesarean section (or vaginal delivery, if possible) at 36 weeks (after documentation of fetal lung maturity) or sooner, if necessary, for patients with a resolved episode of antepartum hemorrhage due to placenta previa or abruption.

The level and intensity of care required is not feasible for most developing countries. Additionally, there are currently no established guidelines or effectiveness data concerning the management of antepartum hemorrhage using a less vigilant approach in resource-poor settings.

**Sepsis**

**Incidence and case fatality rate**

Globally, puerperal sepsis and infection are estimated to contribute to nearly 10% of all maternal deaths in Africa (9.7%), Asia (11.6%), as well as Latin America and the Caribbean (7.7%). Initial estimates for the overall incidence of puerperal sepsis were created using data from the 2002 edition of the WHO’s Global Burden of Disease study. We base our estimates for the risk of puerperal sepsis on the 2000 GBD estimates, that births occurring inside facilities with SBA were assumed to have a risk of puerperal sepsis of 2.5%. We assumed skilled birth attendants adhere to clean delivery practices, and therefore home deliveries attended by SBA had the same risk of puerperal sepsis. Those delivering at home with an untrained attendant had double the risk, at 5.0% . To account for the uncertainty in our initial estimates, we established a range of 4.2% - 6% for sensitivity analysis.

A 2004 Cochrane Review that assessed the effectiveness and safety of antibiotic prophylaxis in reducing infectious puerperal morbidities in women undergoing operative vaginal deliveries. While there was no statistically significant difference in the group of women that was given antibiotics versus those not given antibiotics, there was a relative risk reduction of 93% in the prophylactic antibiotic group . In addition, two studies by Mosha et al. and Winani et al. concluded that women who bathed before delivery and women who used a clean delivery kit were 2.6 and 3.2 times less likely to develop puerperal sepsis than women who did not, respectively. Other studies reported a non-significant difference or inconclusive difference in effectiveness of interventions to prevent puerperal sepsis.

The initial estimate for the case fatality rate (CFR) was from a review conducted by the Disease Control Priorities Project (DCP2) that reported an average CFR of 1.3% and severe sepsis CFR of 3.9%. These estimates were then adjusted for heterogeneity in severity and underlying morbidity (e.g., severe anemia) by calibrating the model to fit multiple epidemiologic targets simultaneously. [See section above on “*Adjustment of case fatality rates for heterogeneity in severity and co-morbidity*”]. Our adjusted CFR widened the implied plausible range. While the literature-based range was 0.009–0.017 (average = 0.013), the expanded plausible range was 0.009–0.036 (average = 0.028). Model-projected mortality due to sepsis, as well as MMR, TFR, and calendar deaths for 2005, closely approximated the empiric data.

**Reduction in mortality**

We assumed the treatment regimen for puerperal sepsis (viz. 2-day intravenous course of Ampicillin, Gentamycin, and Metronidazole followed by an 8-day course of intramuscular Gentamycin and oral Metronidazole) had an overall treatment efficacy of 90% . A similar estimate was used in a recently published modeling analysis conducted by Pagel et al. Assuming a post-partum sepsis CFR of 11% in sub-Saharan Africa, an 8-fold higher sepsis CFR without antibiotics compared to with antibiotics, and a 40% rate of antibiotic use, Pagel et al. estimated an 87.6% reduction in mortality from sepsis. We varied this estimate from 63% to 93% for effectiveness expected in an appropriate EmOC facility.

**Obstructed labor:**

**Incidence and case fatality rate**

The two major causes of obstructed labor are cephalopelvic disproportion and abnormal fetal presentation (e.g., breech or brow presentation). Foremost complications of obstructed labor include endometritis, fistula formation (rectovaginal and/or vesicovaginal) and ruptured uterus with consequent hemorrhage, shock or death. If the obstruction cannot be resolved by manipulation (i.e. to reposition the fetus in the case of abnormal presentation) or instrumental vaginal delivery (with forceps or vacuum), cesarean section is required. Globally, obstructed labor is estimated at 4.6% of live births, although this varies considerably among different regions of the world .

Women who are malnourished, who marry young and/or engage in childbearing at an early age before the pelvis has reached adult proportions, are at high risk for obstructed labor. However, there is no evidence to show that interventions aimed at providing adequate childhood nutrition or delayed childbearing prevents obstructed labor . Using 2002 GBD data we estimated the incidence of obstructed labor. To account for the uncertainty in our initial estimates, we established a range of 3% - 7% for sensitivity analysis.

The initial estimate for the case fatality rate (CFR) was from a review conducted by the Disease Control Priorities Project (DCP2) that reported an average CFR of 0.7%; this estimate was adjusted for heterogeneity in severity and underlying morbidity (e.g., severe anemia) by calibrating the model to fit multiple epidemiologic targets simultaneously [see section above on *Adjustment of case fatality rates for heterogeneity in severity and co-morbidity*]. The adjusted CFR widened the implied plausible range: while the literature-based range was 0.005 – 0.009 (average = 0.007), the expanded plausible range was 0.005 – 0.025 (average = 0.019). Model-projected mortality due to obstructed labor, as well as MMR, TFR, and calendar deaths for 2005, closely approximated the empiric data.

**Reduction in mortality**

We identified multiple studies, including two Cochrane reviews that examined the efficacy of treating obstructed labor in reducing of maternal mortality rates . Yarrow et al. showed a 94.1% success rate when conducting vacuum-assisted deliveries . Of the nine failed vacuum deliveries, four deliveries were subsequently conducted by use of forceps and five by cesarean section; with no maternal mortality was reported. We assumed a 95% reduction in maternal mortality when obstructed labor was managed in an appropriate facility (assisted vaginal delivery with forceps or vacuum and, if necessary, cesarean section). To account for the uncertainty in our initial estimates, we established a range of 76% - 100% for sensitivity analysis.

**Severe pre-eclampsia and eclampsia:**

**Incidence and case fatality rate**

Hypertensive disorders of pregnancy refer to a range of conditions associated with high blood pressure, proteinuria and, rarely, seizures. Severe pre-eclampsia and eclampsia have the highest case fatality rates of the hypertensive disorders of pregnancy, and can lead to placental abruption, disseminated intravascular coagulopathy (DIC), adult respiratory distress syndrome (ARDS), cerebral hemorrhage, seizures, and death. Globally, the incidence of pre-eclampsia is estimated at 3.2% of live births and eclampsia at 0.5% . Eclampsia has a high case fatality rate, which varies among regions of the world, presumably as a function of the access to and quality of health care.

A retrospective study of pre-eclampsia- and eclampsia-related deaths found that access to and delay in seeking care was a major determinant of mortality, with 37.7% in grade IV coma and 54% with recurrent convulsions prior to admission . Initial estimates for the overall incidence of hypertensive diseases of pregnancy were based on data from the 2002 edition of the WHO Global Burden of Disease study. To account for the uncertainty in our initial estimates, we established a plausible range based on our literature review, including studies in the Cochrane database. The only interventions shown to prevent pre-eclampsia are anti-platelet agents, primarily low-dose aspirin, and calcium supplementation. While data from trials are insufficiently conclusive as to the optimal timing of delivery with pre-eclampsia, there is robust evidence that magnesium sulfate can prevent and control eclamptic seizures, and for pre-eclampsia, reduces the risk of eclampsia by more than 50%.

The initial estimate for the case fatality rate (CFR) was from a review conducted by the Disease Control Priorities Project (DCP2) that reported an average CFR of 1.7%; this estimate was adjusted for heterogeneity in severity and underlying morbidity (e.g., severe anemia) by calibrating the model to fit multiple epidemiologic targets simultaneously [see section above on *Adjustment of case fatality rates for heterogeneity in severity and co-morbidity*]. The adjusted CFR widened the implied plausible range. While the literature-based range was 0.012 – 0.022 (average = 0.017), the expanded plausible range was 0.012 – 0.027 (average = 0.021). Model-projected mortality due to hypertensive disorders, as well as MMR, TFR, and calendar deaths for 2005, closely approximated the empiric data.

**Reduction in mortality**

We assumed that severe pre-eclampsia and eclampsia required treatment with intravenous hydralazine and magnesium sulfate; in addition, approximately 10% of all cases were assumed to require emergent cesarean section . A Cochrane Review of magnesium sulphate and other anticonvulsants for women with pre-eclampsia compiled evidence from 6 trials, of which the largest source of data was the Magpie Trial Collaborative Group. This review found a 59% reduction in risk of eclampsia in women with pre-eclampsia (RR 0.41) and a 46% reduction (RR 0.54) in the risk of dying in women with pre-eclampsia randomized to magnesium sulfate . Another review showed that magnesium sulphate was the better anticonvulsant choice when treating women with eclampsia, and substantially reduced the risk of further seizures when compared to diazepam. One goal of this study was whether induction of labor in women with pregnancy induced hypertension or pre-eclampsia at term reduced costs and improved quality of life as compared to expectant monitoring. Two studies looked only at mild pre-eclampsia and gestational diabetes, but not at cases of maternal or neonatal death or eclampsia. A study by one collaborative group found that the use of magnesium sulphate for women with pre-eclampsia was associated with a 16% reduction in the risk of death or serious morbidity related to pre-eclampsia 2 to 3 years later.

We assume that aside from the use of magnesium sulphate, induction of labor could occur in facilities capable of basic and comprehensive emergency obstetric care for women who do not require emergency cesarean section. Thus we rely on the higher effect size from the Cochrane Review, although still perhaps a conservative estimate, for the reduction in the case fatality rate of severe pre-eclampsia. We assumed severely pre-eclamptic/eclamptic women who received treatment had a 59% reduction in disease-specific mortality compared to those without treatment. A Cochrane Protocol for additional evaluation of interventions for treating pre-eclampsia and its complications have been submitted and will be useful for further updating of this estimate once the review has been published. To account for the uncertainty in our initial estimates, and based on the literature review, we established a range of 45% - 95% for sensitivity analysis.

| **Table 12:** Pre-eclampsia and eclampsia | | | | | | | |
| --- | --- | --- | --- | --- | --- | --- | --- |
|  | **Relative Risk (RR)** | | | | **% Reduction in Risk** | | |
|  | RR | 95% CI | |  | % | Lower | Upper |
| Hypertensive disorders of pregnancy (including eclampsia) |  |  |  | | 76% | 71% | 95% |
| Risk of eclampsia among women with pre-eclampsia |  |  |  | | 58% | 40% | 71% |
| Risk of eclampsia in women with pre-eclampsia | 0.41 | (0.29 – 0.58) | | | 59% | 42% | 71% |
| Maternal mortality among women with pre-eclampsia | 0.55 | (0.26-1.14) | | | 45% | 14% | 74% |
| Maternal mortality in women with pre-eclampsia | 0.54 | (0.26, 1.10) | | | 46% | 10% | 74% |
| Death (potentially related to) pre-eclampsia 2-3 years after delivery | 0.84 | (0.60, 1.18) | | | 16% | 18% | 40% |
| Mild pre-eclampsia |  |  |  | | 64% |  |  |

| **Table 13:** Long-term morbidity | | |
| --- | --- | --- |
| **Initial estimates for complication (GBD data)** | |  |
| Neurological sequelae | | 0.0008 |
| Severe anemia^a^ | | 0.090 |
| Sheehan’s syndrome | | 0.008 |
| Infertility from sepsis^b^ | | 0.086 |
| Fistula^c^ | | 0.022 |
| a. | This estimate takes into account the rate of severe and moderate anemia, the overall incidence of PPH, and the overall incidence of severe anemia in pregnant women, during the postpartum period, and in the general reproductive age group. We assume that women with pre-existing moderate anemia contribute disproportionately to the subsequent severe anemia observed following PPH. | |
| b. | Represented by the risk of PID (0.40) multiplied by the risk of infertility (0.22) given PID, to yield the estimate of 0.086 | |
| c. | We assume only 25% are treated but vary this from 25% to 75% in sensitivity analysis. We would seek to use region-specific estimates here. | |

**Maternal deaths due to unsafe abortion**

The World Health Organization (WHO) defines “unsafe abortion” as “the termination of an unintended pregnancy either by persons lacking the necessary skills or in an environment lacking the minimal medical standards, or both.”

The consequences of lack of access to safe abortion in Nigeria are formidable. The procedure is illegal except in cases where the woman's life or health is at risk and this has prompted pregnant women and girls to seek the procedure from people who have not been trained to perform such procedures. In some cases, these untrained providers give the pregnant women herbs before performing abortions, which may be done by punching the pregnant women's stomachs or inserting objects into the vagina and uterus. Local hospitals in Nigeria often have to correct mistakes made by the untrained abortion providers. Because most abortions in Nigeria are performed illegally, there are no reliable abortion figures for the country. Furthermore, as abortion is not available on request, contraception use is low and maternal mortality is high. One study found that 30% of women seeking abortion care had first tried to stop the pregnancy themselves, or had turned to an untrained person. Another study found that 28% had had at least one unwanted pregnancy; 58% of these had attempted to terminate a pregnancy, and 84% of women who attempted had succeeded.

| **Table 14.** Estimates used for sensitivity analysis and stepwise increases in availability and access to safe abortion services. | | | |
| --- | --- | --- | --- |
| **Range of baseline estimates for status quo used in sensitivity analysis** | | | |
|  | **Nationwide** | **Southwest zone** | **Northeast zone** |
| Coverage of safe abortion services | 15% – 30% | 30% - 75% | 5% - 25% |
| **Lower and upper bounds of stepwise increases in availability and access to safe abortion services.** | | | |
|  | **Nationwide** | **Southwest zone** | **Northeast zone** |
| Coverage of safe abortion services | 25% - 100% | 70% - 100% | 10% - 100% |

To estimate the risk of mortality from safe abortion the majority of our estimates were drawn from U.S. data in the early 1970s, a period in which elective first-trimester abortion was legalized in most U.S. states. Data from the Joint Program for the Study of Abortion (JPSA) show that manual vacuum aspiration (MVA) is associated with fewer complications (total and major) compared with dilation and curettage (D&C) , consistent with results from other US large, prospective studies and smaller scale studies in developing countries. By synthesizing British data for 439,400 legal, first-trimester abortions performed between 1968-1973 and U.S. abortion surveillance data from 1972-1975, we estimated the mortality risk for D&C was 1.8 per 100,000 procedures and MVA was 1.3 per 100,000 procedures. Our estimated mortality risk associated with MVA falls within the range of values (1.0 to 1.6 per 100,000 procedures) reported in published literature from the 1970s ; however, we acknowledge the actual risk may be higher in developing countries. For sensitivity analyses on the complications and costs of medical safe abortion, we used data on vaginal misoprostol. We conservatively assumed a success rate of 80% for the vaginal misoprostol regimen, which falls roughly midway between reported estimates: Faundes et al. reported a success rate of 65%-93% for regimens using 800 mcg vaginal misoprostol (1 to 5 doses); Carbonell et al. found a success rate of >90% with 2400 mcg of misoprostol or in very early pregnancies terminated up to 9 weeks gestational age, compared to <90% in those terminated in the late first-trimester. Major complications resulting from medical abortion include pelvic infection and hemorrhage necessitating transfusion and were estimated to arise in 0.75% of all procedures.

**Subsection B**

**Data: Coverage Inputs and Selected Services**

Coverage rates of skilled birth attendants and traditional birth assistants as well as of facility-based births were derived from national databases and published literature. In the model, delivery setting is differentiated by site including (1) home; (2) birthing center or health center (used interchangeable here), (3) facility with bEmOC, (4) facility with cEmOC, and differentiated by health provider including (1) family member, (2) traditional birth attendant [TBA], (3) skilled birth attendant. Facilities classified as birthing centers or health centers are assumed to be staffed by SBA with expectant management of labor but do not have all signal functions to qualify as bEmOC.

**Delivery**

Coverage rates of skilled birth attendants, traditional birth assistants and facility-based births were derived from national databases and published literature. Using data from NDHS-2008, while almost two third of births occur at home, less than 40% of deliveries are attended to by a skilled provider, with a major regional differences, and between rural and urban areas. In the model, delivery setting is differentiated by site [including (1) home; (2) birthing center/health center, (3) facility with bEmOC, (4) facility with cEmOC] and differentiated by health provider [including (1) family member, (2) traditional birth attendant [TBA], (3) skilled birth attendant]. Facilities classified as birthing centers or health centers (used interchangeably here) are assumed to be staffed by SBA with expectant management of labor but do not have all signal functions to qualify as bEmOC.

| **Table 15:** Selected Model Parameters (Nigeria DHS 2008) | | | | |
| --- | --- | --- | --- | --- |
| **Delivery location** | | **National** | **Southwest zone** | **Northeast zone** |
| Total skilled delivery | | 38.9 | 76.5 | 15.5 |
| % begun in facilities | | 35.0 | 70.0 | 12.8 |
| % home delivery (SBA)^a,b^ | | 7.1 | 33.6 | 3.3 |
| a | SBA: skilled birth attendant |  |  |  |
| b | We calculated the percentage of births with skilled attendance at home by subtracting the percentage delivered in facilities (of which 98.5% were attended by skilled personnel) from the total of births with skilled attendance (total skilled delivery – facility based births) / proportion of home births. | | | |

Extrapolating from data on the availability of emergency obstetric services and the relative availability of bEmOC to cEmOC, we estimated for the status quo and found that approximately 30% of facility-based births occur in an EmOC-capable site, of which 17% offer cEmOC. We therefore assume in the base case analysis that for births that occur in a facility with EmOC capacity, approximately 90% are assumed to occur in bEmOC facilities and 10% in cEmOC facilities. In strategies that shift home births to facilities, additional analyses are conducted using several alternative assumptions. For example, as shown below, we explore in a scenario analysis the impact of (a) changing the distribution of routine deliveries that occur in primary facilities lacking EmOC (birthing centers/health centers) and facilities with EmOC, and (b) changing the distribution of deliveries in EmOC that occur in bEmOC versus cEmOC.

**Antenatal care**

The major objective of antenatal care is to identify and treat problems during pregnancy such as anemia and infections. Antenatal care (ANC) visits include screening for complications, and advising on a range of issues such as place of delivery and referral of mothers with complications. Most pregnant women in Nigeria received antenatal care from skilled personnel (22.9 percent by a doctor, 30 percent from a nurse or midwife, and 4.9 percent from an auxiliary nurse of midwife). Data from the Nigerian DHS 2008 shows that expectant mothers are more likely to receive antenatal care from a skilled provider in certain circumstances: if she is aged 20 years and above; if the current pregnancy is her first, second or third; if she resides in an urban area; if she is more formally educated and if she belongs to a higher wealth quintile. Furthermore, women in the South of the country are more likely to receive antenatal care from a skilled provider than women in the North of the country. However, 36% did not receive antenatal care.

Data on antenatal care (ANC) from the **Nigeria DHS 2008** were initially used for coverage rates for the national model, and were stratified by urban and rural status when available. We assumed in our analyses that antenatal care includes 4 visits, tetanus vaccination, syphilis, gonorrhea, Chlamydia screening (and treatment), urinalysis, blood tests, treatment for anemia, counseling (e.g., family planning, spacing, intrapartum care).

| **Table 16:** National and state-specific ANC and anemia treatment rates (Nigeria DHS 2008) | | | | |
| --- | --- | --- | --- | --- |
|  | **% that had 4 or more**  **ANC visits** | **% with an ANC visit in the 1^st^ trimester of pregnancy** | **Median months pregnant at first ANC visit** | **% who took iron supplements** |
| Nigeria | 44.8 | 16.2 | 5.0 | 54.3 |
| Urban | 68.8 | 22.2 | 5.0 | 77.4 |
| Rural | 34.4 | 13.7 | 5.1 | 44.4 |

| **Table 17:** Prenatal care and treatment of anemia | | | |
| --- | --- | --- | --- |
| **Interventions** | **Nigeria** | **Southwest zone** | **Northeast zone** |
| Prenatal care (%) | 57.7 | 87.1 | 43.0 |
| Treatment of anemia (%) | 54.3 | 87.8 | 46.0 |

| **Table 18:** Sensitivity analysis exploring the benefits of universal antenatal care in isolation, when combined with increasing facility birth and increasing cEmOC birth. | | | |
| --- | --- | --- | --- |
|  | **Analysis 1** | **Analysis 2** | **Analysis 3** |
| **Prenatal care** | Vary 35% - 100% | Vary 35% - 100% | Vary 35% - 100% |
| **Facility birth** | Vary 30% - 100% | Vary 30% - 100% | Vary 30% - 100% |
| Analysis 1= 90% bEmOC:10% cEmOC; Analysis 1 = 50% bEmOC:50% cEmOC; Analysis 3 = 10% bEmOC:90% cEmOC | | | |

**Family planning**

Our choice of including a comprehensive strategy of enhanced family planning is to reduce the unmet need for contraception, for purposes of *both* limiting and spacing. The effectiveness of family planning is incorporated into the model as a set of variables that reflect several parameters viz.: coverage level of contraceptive method; distribution of contraceptive type; type-specific failure rate. We use state-level and setting-specific data to represent the current met need for contraception and the distribution of methods used by age. Failure rates are conditional on the method used. Estimates relating to unmet need for family planning are also from the Nigeria Demographic and Health Survey 2008**.** Approximately 20 percent of currently married women in Nigeria have an unmet need for family planning, with limiting (5%) around one-third that of spacing (15%) using average national estimates. Women aged 15-24 have a greater unmet need for spacing than for limiting. Women in rural Nigeria have a higher unmet need for spacing but lesser unmet need for limiting relative to their urban counterparts. In our base case analysis both age-patterns and rural/urban status are incorporated.

| **Table 19:** Coverage level of contraceptive methods (Nigeria DHS 2008) | | | | | |
| --- | --- | --- | --- | --- | --- |
|  | | **Baseline Value (%)** | | | |
| **Family planning** | | **Nigeria ^b^** | **Nigeria ^c^** | **Urban ^c^** | **Rural ^c^** |
| Any method | | 15.4 | 14.6 | 25.9 | 9.4 |
| Modern methods | | 10.5 | 9.7 | 16.7 | 6.5 |
|  | Pill | 15.2 | 17.5 | 19.8 | 15.4 |
|  | IUD | 6.7 | 10.3 | 13.2 | 6.2 |
|  | TOL | 2.9 | 4.1 | 2.4 | 6.2 |
|  | Injectables | 19.0 | 26.8 | 22.2 | 30.8 |
|  | LAM | 10.5 | 16.5 | 13.2 | 20.0 |
|  | Implants | 0.0 | 0.0 | 0.6 | 0.0 |
|  | Male condoms | 0.0 | 0.0 | 28.7 | 21.5 |
|  | | |  |  |  |
| **Family planning** | | | **Nigeria ^b^** | **Southwest zone ^c^** | **Northeast zone ^c^** |
| Any method | | | 15.4 | 31.7 | 4.0 |
| Modern methods | | | 10.5 | 21.0 | 3.5 |
|  | Pill | | 15.2 | 4.0 | 0.6 |
|  | IUD | | 6.7 | 3.1 | 0.0 |
|  | TOL | | 2.9 | 0.2 | 0.2 |
|  | Injectables | | 19.0 | 4.5 | 0.9 |
|  | LAM | | 10.5 | 3.0 | 1.5 |
|  | Implants | | 0.0 | 0.0 | 0.0 |
|  | Male condoms | | 0.0 | 6.1 | 0.2 |
|  |  | | | | |
| a | IUD: intrauterine device; TOL: female sterilization | | | | |
| b | All women included | | | | |
| c | Currently married women only | | | | |

| **Table 20:** Failure rates of contraceptive methods (UNFPA 2007) | |
| --- | --- |
| **Contraceptive method** | **Failure rates** |
| Male sterilization | 0.2% |
| Female sterilization | 0.5% |
| Injectable contraceptives | 2.9% |
| Intrauterine device | 4% |
| Oral contraceptives | 8% |
| Condoms | 19% |

**Overview of our strategies to increase contraceptive use**

We elected to use unmet need as our main intervention target to describe the influence of increasing access and uptake of modern contraception, as it reflects two groups of women: (a) women who are not using any method of contraception but who do not want any more children - unmet need for *limiting* and (b) those who are not using contraception but want to wait two or more years before having another child - unmet need for *spacing* (the sum of the unmet need for limiting and the unmet need for spacing is the modeled unmet need for family planning).

| **Table 21:** Changes in unmet need for contraception nationwide, and in southwest and northeast zones. | | | | | | | | | |
| --- | --- | --- | --- | --- | --- | --- | --- | --- | --- |
|  | **Nationwide** | | | **Southwest zone** | | | **North east zone** | | |
|  | For spacing | For limiting | Total unmet need | For spacing | For limiting | Total unmet need | For spacing | For limiting | Total unmet need |
| 2003 DHS | 11.8% | 5.1% | 16.9% | 11.3% | 5.9% | 17.2% | 13.1% | 0.5% | 13.6% |
| 2008 DHS | 15.0% | 5.2% | 20.2% | 12.6% | 7.1% | 19.7% | 13.6% | 0.4% | 14.0% |
| DHS = Nigerian Demographic and Health Survey. | | | | | | | | | |

For our primary analysis, we reduced the unmet need for contraception by 25% to 100% for both the national and zonal analyses. Anticipating changes to fertility preferences over time, we conducted a secondary analysis in which the use of modern contraceptives was increased by 25% to 50%, nation wide and in both southwest and northeast zones.

| **Table 22**: Different approaches used in increasing the availability of modern methods of contraception in the model. | | | | | | | | | |
| --- | --- | --- | --- | --- | --- | --- | --- | --- | --- |
|  |  | **Primary analysis of family planning** | | | | **Secondary analysis of family planning** | | | |
|  | **Status quo** | **Reducing the unmet need for contraception** | | | | **Increasing use of modern contraceptives** | | | |
|  |  | **by 25%** | **by 50%** | **by 75%** | **by 100%** | **by 25%** | **by 30%** | **by 40%** | **by 50%** |
| National | 9.7% | 14.8% | 19.8% | 24.9% | 29.9% | 34.7% | 39.7% | 49.7% | 59.7% |
| Southwest zone | 21.0% | 24.6% | 28.1% | 31.7% | 35.2% | 46.0% | 51.0% | 61.0% | 71.0% |
| Northeast zone | 3.5% | 7.4% | 11.3% | 15.1% | 19.0% | 28.5% | 33.5% | 43.5% | 53.5% |
| For the national analysis, the status quo and increments were applied in an age-specific manner. For the zonal analyses, average values were used. Status quo data was obtained from the 2008 Nigeria Demographic and Health Survey. | | | | | | | | | |

**Postpartum care**

While it was difficult to find quantitative data on reduction in mortality with postpartum care, but we did take the data from the DHS into account in the model.

| **Table 23:** Maternal care indicators in the Southwest and Northeast zones (Nigeria 2008 DHS) | | | |
| --- | --- | --- | --- |
|  | | **Percent Deliveries** | |
|  | | **With postnatal check-up ^a^** | **With postnatal check-up within 2 days** |
| Nigeria | | 41.5 | 38.3 |
| Southwest zone | | 73.1 | 67.6 |
| Northeast zone | | 29.2 | 27.1 |
| a | Based on the last birth within the five years preceding the survey. Postnatal check-ups are defined as checks on the woman's health within 42 days of delivery. | | |

**Data and Assumptions: Barriers to Effective Referral**

**Barriers to Effective Referral to EmOC**

Effective referral relies on the ability to overcome three critical delays (a) recognition of referral need and willingness to be referred (by provider and delivery location); (b) expedient transfer to referral facility (determined by distance, affordability, available transport); and (c) timely treatment in an appropriate facility capable of high-quality emergency obstetrical care (e.g., 6 signal functions in bEmOC, blood transfusion and surgery in cEmOC). We expanded Thaddeus and Maine’s "three delays" framework to reflect the multidimensional nature of each of these delays and the heterogeneity between and within countries as to which delays and components are most critical. A successful referral in our model incorporates a series of elements, each of which could act as a barrier to the care a woman with pregnancy-related complications requires.

**Delay Category 1. Recognition of need for referral and/or willingness to be referred**

We include in this category both failure or delay in recognition of the need for referral by the SBA as well as delay in recognition for need of referral or unwillingness to be referred on the part of the woman or her family. We assumed the recognition rate for complications developing during home deliveries would vary based on the level and skill of the birth attendant. Based on data from Honduras regarding traditional (untrained) birth attendants, we assumed an 11.5% recognition rate for unskilled delivery at home, and a 20% recognition rate for skilled birth attendants at home. Based on literature and Nigeria-specific data, we established a plausible range for sensitivity analysis. We assumed life-threatening complications (those needing cEmOC capability) occurring at bEmOC were recognized as needing transfer to a facility with cEmOC. We also included an analysis assessing the impact of delays in facility transfers (i.e., incorporating the delay due to transport problems, logistics, or fees). In addition, we assumed an “erroneous” referral rate (in the absence of complications), owing to misdiagnosis and lack of patient monitoring support, that varied from 2.5% to 10% based on location of delivery and skill level of birth attendant.

**Rationale for assumptions used in Nigeria model**

A close look at the 2008 DHS reveals that although 58% of women within the reproductive age group had contact with health professionals during pregnancy, less than two-thirds of them received advice about danger signs and about one-third delivered in a health facility. Furthermore, three-quarters of the female respondents in this age group had at least one problem in accessing healthcare; 14% required permission, 56% lacked adequate funds to pay for treatment, 36% lived too far away and 34 % had transportation difficulties. These delays add to the burden of maternal mortality. Orji et al reported the following of brought-in maternal deaths in Southwest Nigeria:

- 1. 42% resulted from inability to obtain transportation in time
  2. 25% resulted from unwillingness of drivers to travel at night
  3. 33.3% resulted from late recognition of an obstetric emergency and late referral by the referring hospital
  4. 33.3% resulted from the inability of the staff at the referring hospital to perform an emergency Caesarian Section.
  5. 25% resulted from fear of Caesarian section and
  6. 16.7% resulted from inability to pay for hospital costs.

| **Table 24:** World Bank HNP Database 2008 Maternal Health *by Rural Urban Status* | | | | | | |
| --- | --- | --- | --- | --- | --- | --- |
| **Status of women in Nigeria** | Rural | | | Urban | | |
|  | Low | High | Avg. | Low | High | Avg. |
| **Household decision making:** |  |  |  |  |  |  |
| Can seek own health care | 14.4 | 17.2 | 13.7 | 18.6 | 22.4 | 19.6 |
| **Other decision making, attitudes:** |  |  |  |  |  |  |
| Can decide how to spend own money | 67.1 | 67.8 | 70.8 | (63.8) | 79.4 | 77.8 |
| Can decide whether to have sex | 86.2 | 89.0 | 86.7 | 78.8 | 90.5 | 89.9 |

| **Table 25:** World Bank HNP Database 2008 Maternal Health *by Wealth* | | | | | |
| --- | --- | --- | --- | --- | --- |
| **Status of women in Nigeria** | Wealth Quintiles | | | | |
|  | Low | 2nd | 3rd | 4th | High |
| **Household decision making:** |  |  |  |  |  |
| Can seek own health care | 14.6 | 10.1 | 13.4 | 18.6 | 21.0 |
| **Other decision making, attitudes:** |  |  |  |  |  |
| Can decide how to spend own money | 66.9 | 71.8 | 76.0 | 74.3 | 76.5 |
| Can decide whether to have sex | 85.9 | 87.5 | 86.0 | 89.0 | 90.1 |

The Tables below show the initial *range* of baseline estimates that will be used in sensitivity analyses, and the initial *range* across which stepwise improvements will be made in the temporal strategies evaluated.

| **Table 26.** Estimates used for sensitivity analysis and stepwise increases in recognition of referral need. | | | |
| --- | --- | --- | --- |
| **Range of baseline estimates for status quo used in sensitivity analysis** | | | |
| Recognition of referral need | **Nationwide** | **Southwest zone** | **Northeast zone** |
| Unskilled at home | 8% - 20% | 8% - 20% | 8% - 20% |
| Skilled at home | 15% - 30% | 15% - 30% | 15% - 30% |
| Skilled at birthing center ^a^ | 30% - 60% | 30% - 60% | 30% - 60% |
|  |  |  |  |
| **Lower and upper bounds of stepwise increases in recognition of need/willingness for referral** | | | |
| Recognition of referral need | **Nationwide** | **Southwest zone** | **Northeast zone** |
| Unskilled at home | 8% - 30% | 8% - 30% | 8% - 30% |
| Skilled at home | 20% - 90% | 20% - 90% | 20% - 90% |
| Skilled at birthing center ^a^ | 40% - 95% | 40% - 95% | 40% - 95% |
| A birthing center not considered EmOC; we consider all attendants at EmOC to be skilled. | | | |

**Delay Category 2. Expedient transfer from birth location to facility**

In this category, we include the availability of the following: a timely and affordable means of transportation from birthing location to facility; a functioning vehicle with fuel; and the provision of interim lifesaving care en route. The availability of transport is assumed to be a function of infrastructure (ambulances, neighborhood emergency transport networks, road densities, distance to hospitals, etc. The geography of Nigeria, its size, diversity and dispersion give roads a special place in integration of the national economy. Although roads serve rural areas (where 67% of the populace reside) more effectively than any other mode of transport, national networks face lack of sufficient fund for rehabilitation and routine maintenance. This is in the setting of low capacity of the local construction industry and low participation of the private sector. However, the Government has streamlined the institutional framework for management of the road sector so as to enhance efficiency, effectiveness and accountability. We made assumptions about effective transfer that varied by delivery location, and were intended to reflect access to transport, reliable fuel and accompanying person en route, and interim care if necessary. The Tables below show the initial *range* of baseline estimates that will be used in sensitivity analyses, and the initial *range* across which stepwise improvements will be made in the temporal strategies evaluated. These ranges will be expanded in a series of exploratory analyses.

| **Table 27**: Average travelling distance to nearest primary healthcare centers by zone | | | | |
| --- | --- | --- | --- | --- |
| **ZONE** | **Average distance travelled in rainy season** | | **Average distance travelled in dry season** | |
| National | 23 km | (14.3 miles) | 23 km | (14.3 miles) |
| South West | 14 km | (8.7 miles) | 13 km | (8.1 miles) |
| North East | 32 km | (19.9 miles) | 33 km | (20.5 miles) |
| WHO recommended | ≤ 10 km | (≤ 6 miles) | ≤ 10 km | (≤ 6 miles) |

| **Table 28.** Range of baseline estimates for status quo used in sensitivity analysis | | | |
| --- | --- | --- | --- |
| Expedient transfer to an appropriate referral facility | **Nationwide** | **Southwest zone** | **Northeast zone** |
| From home | 30% - 40% | 50% - 70% | 20% - 35% |
| From birthing center | 30% - 40% | 50% - 70% | 20% - 35% |
| From bEmOC ^a^ | 30% - 40% | 50% - 70% | 20% - 35% |
| **Lower and upper bounds of stepwise increases in ability to transfer to an appropriate referral facility.** | | | |
| Expedient transfer to an appropriate referral facility | **Nationwide** | **Southwest zone** | **Northeast zone** |
| From home | 30% - 95% | 50% - 95% | 25% - 95% |
| From birthing center | 30% - 95% | 50% - 95% | 25% - 95% |
| From bEmOC ^a^ | 30% - 95% | 50% - 95% | 25% - 95% |
| Estimates used for sensitivity analysis and stepwise increases in ability to transfer to an appropriate facility expediently, reflecting transport, supportive care en route, and no delays). a – in a situation where cEmOC would be necessary for lifesaving transfusion, surgery or management of shock. | | | |

**Delay Category 3. Availability and quality of services at EmOC facilities**

We include in this category availability and quality of services at EmOC facilities, including the presence of a facility open 24 hours per day with adequate staffing and supplies, expedient attention (e.g., without delay to collect fees or requirement for family to bring supplies), and care that is evidence-based and of high-quality. Assumptions for this category are challenging as even in locations where there might be adequate numbers of doctors, or an adequate number of facilities, attributes such as round-the-clock availability, expedient care without delay, adequate supplies, and high-quality practice are critically influential on the effectiveness of health service delivery.

The facility categories are flexibly modeled such that particularities of the public health infrastructure in different settings (country, state, rural versus urban areas) can be accurately represented in terms capacity and cost. Facility levels are categorized as (1) *primary- facilities,* which may not have all bEmOC functions but could function as birthing centers with SBA staffing, 24-hour intrapartum care, and reliable referral connections (e.g., sub center, primary health center [PHC]); (2) *secondary facilities* with bEmOC capacity (e.g., first referral unit [FRU], community health center [CHC], General Hospital; and (3) *tertiary facilities* with cEmOC capacity (e.g., district hospital, some first referral units, Teaching Hospitals/Federal Medical Centers).

| **Table 29:**  Framework to differentiate facilities in Nigeria | | | | |
| --- | --- | --- | --- | --- |
| **Health facilities in Nigeria** | **Level** | **Staff** | **Model Category** | **Model Assumptions** |
| Teaching hospitals  Federal Medical Centers  Specialist hospitals | 3^rd^ | Specialists (Ob/Gyn) | Tertiary facility with cEmOC | *Obstetricians:* active-management of labor, blood transfusion, C-section, hemodynamic support |
| General hospitals  Comprehensive Health Centers | 2^nd^ | Med Officers, Specialists (Ob/Gyn) | Tertiary facility with cEmOC | *Obstetricians:* active-management of labor, blood transfusion, C-section, hemodynamic support |
| Primary Health care centers | 1^st^ | Med Officers, Staff Nurse | bEmOC | *SBA:* active-management of labor |
| Health clinics  Health posts | 1^st^ | Female and Male Health Workers | Health center (HC) or birthing center (BC) | *SBA:* expectant-management of labor |
| FCT = Federal Capital Territory also known as Abuja; LGA = Local Government Area). From the National Primary Health Care Development Agency of Nigeria. | | | | |

In the model delivery setting is differentiated by site including (1) home; (2) birthing center or health center (used interchangeable here), (3) facility with bEmOC, (4) facility with cEmOC, and differentiated by health provider including (1) family member, (2) TBA, (3) SBA. Facilities classified as birthing centers or health centers are assumed to be staffed by SBA with expectant management of labor but do not have all signal functions to qualify as bEmOC. Facilities with basic EmOC (bEmOC) are assumed to be capable of administering injectable antibiotics, oxytocics, and sedatives or anti-convulsants, performing manual removal of placenta, removal of retained products, and assisted vaginal delivery. Facilities with comprehensive EmOC (cEmOC) also are able to provide blood transfusion, cesarean section, and management of advanced shock. We recognize that some tertiary sites will not have a blood bank and some secondary sites may eventually be able to perform c-section; further, we recognize that in the strategies that include stepwise investments in infrastructure and facility improvements, not all facilities will be expected to be fully implemented as one of the three distinct types. However, because the costs, functions and staffing are fairly closely aligned with basic or comprehensive EmOC capacity, this simple categorization captured the most important dimensions for purpose of this analysis.

| Table 30: Summary of availability of cEmOC services nationwide and by zone | | | | | | |
| --- | --- | --- | --- | --- | --- | --- |
| **ZONE** | **Use of antibiotics in the Treatment**  **of Sepsis** | **Misoprostol Available** | **Initiation of Treatment of Eclampsia** | **Vacuum Extraction Done** | **Post-Abortal care** | **Functional**  **MVA Set** |
| National | 70% | 30% | 28% | 11% | 37% | 21% |
| South West | 61% | 22% | 6% | 6% | 20% | 22% |
| North East | 78% | 26% | 47% | 14% | 54% | 24% |

| Table 31: Summary of availability of cEmOC services nationwide and by zone | | | | | | | | |
| --- | --- | --- | --- | --- | --- | --- | --- | --- |
| **ZONE** | **Treatment**  **of Sepsis** | **Misoprostol Available** | **Treatment of Eclampsia** | **Vacuum Extraction Done** | **Post-Abortal care** | **Postpartum hemorrhage** | **Blood transfusion service** | **Caesarian section service** |
| National | 92.6% | 50.9% | 87.1% | 38.7% | 85.9% | 89.0% | 88.3% | 84.7% |
| South West | 94.4% | 50.0% | 77.8% | 44.4% | 88.9% | 83.3% | 88.9% | 77.8% |
| North East | 86.1% | 33.3% | 86.1% | 30.6% | 86.1% | 80.6% | 88.9% | 80.6% |

We carried out an in-country survey in May 2011 as a rapid and tactical assessment of the quality of Nigerian healthcare facilities, to help inform assumptions in the model (alongside expert opinion). We visited 121 facilities in the 6 geopolitical zones and some of our findings are presented below (**Table 32**).

| **Table 32:** Some measures of facility quality from a survey we carried out in May 2011 . | | | | | | |
| --- | --- | --- | --- | --- | --- | --- |
|  | **Nationwide** | | **Southwest zone** | | **Northeast zone** | |
| **SERVICE PROVISION** | bEmOC | cEmOC | bEmOC | cEmOC | bEmOC | cEmOC |
| Can always provide emergency obstetric care | 41% | 97% | 40% | 90% | 9% | 89% |
| Can administer injectable antibiotics | 77% | 95% | 100% | 100% | 91% | 100% |
| Can diagnose and treat eclampsia | 23% | 62% | 20% | 70% | 55% | 89% |
| Can conduct assisted vaginal delivery | 10% | 52% | 0% | 50% | 27% | 56% |
| Can diagnose and treat severe shock | 36% | 90% | 20% | 80% | 73% | 100% |
| Can safely transfuse blood | 12% | 90% | 30% | 100% | 9% | 89% |
| Can carry out Caesarian sections | 7% | 82% | 10% | 70% | 9% | 100% |
| **STAFF AVAILABILITY (at least one of the following clinicians is constantly available)** |  |  |  |  |  |  |
| A doctor | 20% | 95% | 20% | 90% | 9% | 100% |
| A nurse/midwife | 48% | 93% | 50% | 80% | 9% | 100% |
| An obstetrician | 8% | 47% | 30% | 70% | 9% | 11% |
| A pediatrician | 5% | 30% | 0% | 60% | 9% | 11% |
| An anesthesiologist | 7% | 47% | 0% | 50% | 9% | 67% |
| **INFRASTRUCTURE** |  |  |  |  |  |  |
| Has an ambulance for transporting pregnant women to referral facilities | 5% | 72% ^a^ | 10% | 89% ^a^ | 9% | 50% ^a^ |
| Has at least one labor ward | 79% | 100% | 80% | 100% | 73% | 100% |
| Has a least one delivery room | 74% | 100% | 80% | 100% | 55% | 100% |
| Has at least one functional operating room | 16% | 95% | 10% | 100% | 9% | 100% |
| Has a neonatal ward/ICU | 10% | 55% | 0% | 60% | 9% | 22% |
| Has uninterrupted electricity supply when there are obstetric emergencies | 15% | 47% | 40% | 90% | 9% | 89% |
| ICU = Intensive Care Unit; n = sample size.  a – applies to only secondary care facilities. | | | | | | |

We conducted both sensitivity analyses on alternative baseline assumptions, as well as an analysis at every stepwise point. The Tables below show the initial *range* of baseline estimates that will be used in sensitivity analyses, and the initial *range* across which stepwise improvements will be made in the temporal strategies evaluated. These ranges will be expanded in a series of exploratory analyses.

| **Table 33.** Estimates used for sensitivity analysis and stepwise increases in the availability of appropriate staff and supplies in EmOC facilities. | | | |
| --- | --- | --- | --- |
| **Range of baseline estimates for status quo used in sensitivity analysis** | | | |
| Appropriate staff and supplies | **Nationwide** | **Southwest zone** | **Northeast zone** |
| bEmOC | 30% - 40% | 20% - 50% | 20% - 50% |
| cEmOC | 50% - 70% | 50% - 70% | 50% - 70% |
| **Lower and upper bounds of stepwise increases in the availability of appropriate staff and supplies at EmOC facilities.** | | | |
| Appropriate staff and supplies | **Nationwide** | **Southwest zone** | **Northeast zone** |
| bEmOC | 30% - 95% | 50% - 95% | 20% - 95% |
| cEmOC | 30% - 95% | 50% - 95% | 20% - 95% |

**Subsection C**

**Calibration Exercises and Model Performance**

Calibration targets for each national and region-specific model are established based on survey data and published studies, and include the distribution of causes of maternal mortality (e.g., PPH, obstructed labor, sepsis), maternal mortality ratio (MMR), and the total fertility rate (TFR). The MMR is adjusted directly in the model for indirect causes of maternal-related mortality, as explained below. The importance of using multiple indicators is that different aspects of maternal mortality are reflected by each of them. For example, the MMR is not age-standardized, nor does it take into account that women face the same risk numerous times over their reproductive lifespan, nor does it account for the reduction in risk attributable to declining fertility from family planning. The model can be used to project a range of maternal health indicators and these can be used as calibration targets, or can be compared to survey data to assess an approximation of face validity or projective validity. These include the following:

| Maternal mortality rate | Defined as the number of maternal deaths per 1,000 women or 100,000 women of reproductive age (ages 15-45) or woman-years of risk exposure, and designed to be an indicator of risk of maternal death (i.e., cause-specific death rate) |
| --- | --- |
| Proportionate mortality ratio | Defined as the proportion of all female deaths among women of reproductive age due to maternal causes |
| Lifetime risk of maternal death | Reflects the probability of a maternal death during a woman’s reproductive lifespan (the probability that a 15-year-old will eventually die from a maternal reason up to age 45, for example) and is described in terms of odds (it accounts for the probability of dying from maternal causes each time a woman experiences a pregnancy, and so takes into account fertility as well as obstetric risk) |
| Lifetime risk of dying from maternal causes | The calculation of lifetime risk assumes no changes in fertility or mortality; estimates are generated from the maternal mortality rate, and do take into account the competing causes of death. In contrast, in our model, the simulation over time does take into account the changes in fertility and background mortality, including changes in maternal mortality. |

**MMR**

We used selected data to inform the Nigeria model MMR calibration target. Below, the range of MMRs using different sources and methods is provided. It is widely accepted that the error and uncertainty in these measures is formidable, and trends should be interpreted with grave caution. As sample sizes decrease, such as at the state or district level, interpretation of trends should be avoided. UNICEF, WHO, and the UN Population Fund (UNFPA) previously developed global, regional, and country estimates of maternal mortality for the years 1990, 1995, and 2000. In 2006 a collaborative effort involving the WHO, UNICEF, UNFPA, the World Bank, and outside technical experts, reviewed the available data and developed revised estimates of maternal mortality for 2005. Countries were categorized on the basis of data availability, quality and type, and consensus was reached on methods of evaluation, data synthesis, and statistical modeling for estimation in countries with limited data. Nigeria was classified as Group H (Group H: Countries who’s MMR was derived from models because they lacked appropriate empirical data) and for 1995–2005 estimated at 1100 (440–2000).

Most recently, a systematic analysis reporting maternal mortality for 181 countries, 1980-2008 was published. Based on a constructed database of 2651 observations from vital registration data, censuses, surveys, and verbal autopsy studies, MMRs were estimated for each year between 1980 and 2008. For Nigeria, these are shown below.

| **Table 34: Published estimates of MMR in Nigeria** | |
| --- | --- |
| **Value (Range)** | **Source** |
| 1100 (440–2000) | Hill 2007 |
| 694 (435-1041) | (2000) Hogan 2010 |
| 608 (372-946) | (2008) Hogan 2010 |

**Distribution of causes of maternal deaths – calibration data**

A systematic review and analysis of the magnitude and causes of maternal deaths has shown variation both across and within geographical regions. Estimates of specific causes of death in Nigeria are hindered by the same methodological challenges as in global estimates. We used Khan et al regional estimates based on the large sample sizes, and took into consideration other data from Nigeria.

| **Table 35: Maternal mortality causes** | | |
| --- | --- | --- |
|  | **Africa (%)** | **CI (%)** |
| Maternal hemorrhage | 33.9 | 13.3 - 43.6 |
| Hypertensive disorders | 9.1 | 3.9 - 21.9 |
| Obstructed labor | 4.1 | 0.0 - 10.3 |
| Sepsis | 9.7 | 6.3 - 12.6 |
| Abortion | 3.9 | 0.0 - 23.8 |
| Subtotal | 60.7 |  |
| Other direct ^a^ | 4.9 | 0.0 - 10.3 |
| HIV/AIDS | 6.2 | 0.0 - 13.3 |
| Anemia ^a^ | 3.7 | 0.0 - 13.2 |
| Other indirect ^a^ | 19.2 | 9.1 - 29.3 |
| Unclassified ^a^ | 5.4 | 0.0 - 21.8 |
| *Total Indirect/Other* ^a^ | *24.6* |  |

The role of anemia in maternal mortality has been previously documented (generally classified as an indirect cause) with reporting 12.5% and Mills et al. reporting 15%). The MMR is adjusted directly in the model for indirect causes of maternal-related mortality. We assume that the proportion of mortality that is categorized as indirect and attributable to anemia will be reduced with strategies that include enhanced family planning, increases in appropriate antenatal care with completed courses of treatment for anemia, facility-based births with quality intrapartum care, and reliable access to basic and comprehensive EmOC. We conservatively assume that the proportion of mortality that is categorized as indirect and attributable to other causes will not be impacted. According to country-specific data in Nigeria the main causes of maternal deaths in 2006 were hemorrhage, sepsis, malaria, anemia, abortion, Eclampsia and CPD (cephalopelvic disproportion from obstructed labor).

| Fig 2: Obstetric and Medical causes of maternal deaths in Nigeria (Sources: National Planning Commission 2001 and Nigerian Health Review 2006) |
| --- |

| 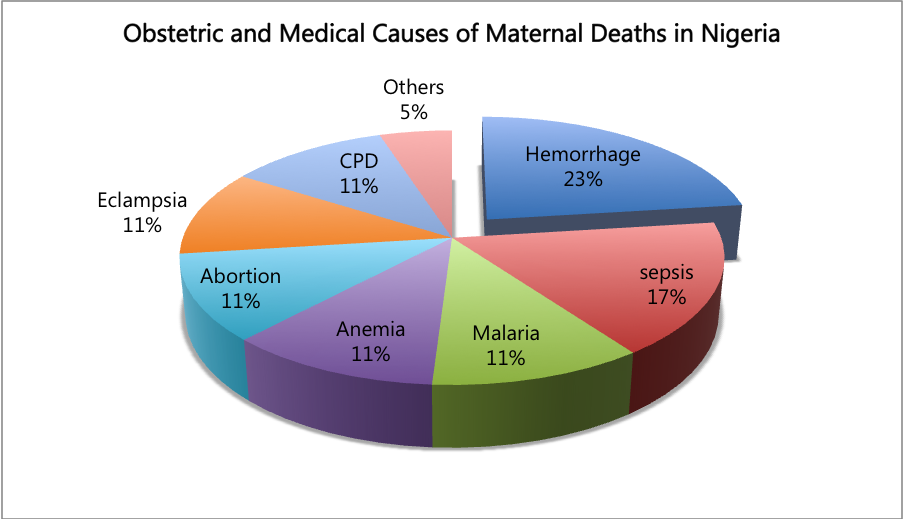 |
| --- |

Preliminary calibration required very minor adjustments across the plausible range established for the initial baseline parameters. These will be revised when the complete analysis is completed with the re-parameterized model.

| **Table 36:** Adjustments to initial estimates of variables in calibration exercises | | | | |
| --- | --- | --- | --- | --- |
| ***Variable*** ^a^ | | **Nationwide** | **Southwest zone** | **Northeast zone** |
|  | Annual probability of pregnancy | 1.0 | 1.0 | 1.0 |
|  | Incidence of PPH | 1.0 | 1.0 | 1.0 |
|  | CFR of PPH | 1.0 | 1.0 | 1.0 |
|  | CFR for life-threatening complications ^b^ requiring transfusion, surgery, management of shock in absence of emergency care | 3.5 – 4.0 | 1.5 – 2.0 | 4.5 – 5.0 |
|  | CFR for unsafe abortion | 1.0 | 1.0 | 1.0 |
| a | PPH: postpartum hemorrhage; CFR: case fatality rate. | | | |
| b | Distribution of severe life threatening complications was adjusted according to basic and comprehensive EmOC need, and is described in section II of this document in the section, “severity of complications”. | | | |

Model performance was assessed by comparison of model-based projections with reported measures such as life expectancy, proportionate mortality ratio, and population-based outcomes. We parameterized the zonal-level models for the southwest and northeast zones using the best information available, and compared projected maternal health indicators from simulation with reported data.

| Table 37: Comparing model output of maternal health indices with those previously published | | | | | |
| --- | --- | --- | --- | --- | --- |
|  | **WHO** | **UNICEF** | **UNFPA** | **World bank** | **Model estimates** |
| MMR | 800 | 840 | 1100 | 840 | 829 |
| TFR | 5.7 | 5.2 | 5.1 | 5.7 | 5.9 |
| Life expectancy | 46 | 48 | 48.8 | 48 | 47 |

| Table 38: Comparing model outputs of direct causes of maternal deaths with estimates from previous studie | | |
| --- | --- | --- |
| **Causes of maternal death** | **Regional Estimates** | **Model output** |
| Unsafe Abortion | 9.8% | 8.8% |
| Eclampsia | 18.5% | 20.0% |
| OL | 8.8% | 14.8% |
| PPH | 48.9% | 38.4% |
| Sepsis | 14.0% | 18.0% |

**PART III: OVERVIEW OF COSTS AND ESTIMATES**

**Overview: cost identification and measurement**

Direct health care costs include the cost of a normal pregnancy (e.g., prenatal visits, normal labor and delivery), the cost of induced abortion, the cost of treating abortion-related complications, the cost of treating Pregnancy-related complications (e.g., eclampsia, hemorrhage, sepsis), salaries of health care providers (e.g., counseling, skilled birth attendants, clinician time); costs related to prenatal care (e.g., additional prenatal visits, nutritional supplementation, treatment of anemia or other existing disease, screening for sexually-transmitted diseases [STDs]), providing safe abortion (e.g., manual vacuum aspiration) or family planning options (e.g., sterilization, intrauterine device [IUD], oral contraceptives), and emergency obstetric care (e.g., facilities with the capacity for transfusion, parental antibiotics, surgery, anesthesia). Direct non-health care costs include, but are not limited to, the costs of transportation to and from the clinic or provider, and costs of patient time seeking care or receiving care. Cost estimates are broken down by input (e.g., drugs, vaccines, salaries, infrastructure), by intervention (e.g., management of a normal birth, hemorrhage, eclampsia, sepsis), and by service location or level (e.g., hospital, health center, health post). Personnel cost (salaries) and facility costs are country-specific from International Labour Organization databases and data publicly available from the World Health Organization (WHO), and modified using state-specific data when available. Salaries originally reported in year 2000 International dollars were converted to local currency units using Purchasing Power Parity conversion rates, inflated to year 2008 local currency units using gross domestic product (GDP) deflators, and then converted to 2008 US dollars using exchange rates. When possible, we conducted literature reviews for costs associated with different services and complications; these costs were extrapolated and adjusted to the same year and currency to facilitate comparison and generate plausible ranges for each cost estimate. Differential costs of scale-up were assessed, as were training costs (e.g., for SBA).

Costs are presented in currency units that remove price inflation, and for analyses intended to inform resource allocation and compare studies from multiple countries, costs are expressed as US dollars or international dollars. While exchange rates may reflect under- or overvaluation of the local currency, they represent what is actually paid for locally produced inputs. Purchasing-power parity rates, in contrast, attempt to express what the local currency is worth in purchasing power, and therefore account for differences in price levels across countries. The exchange rate for domestic currency into international dollars is the amount of domestic currency required to purchase the same quantity of goods and services as $1 could purchase in the US.

**Documentation of costs used in the Nigeria model**

The Nigeria model requires country-specific estimates of all maternal interventions including safe abortion and long-term complications. Estimates in the current model were drawn from the UNFPA’s Reproductive Health Costing Tools Model (RHCTM). This model is designed to help countries estimate the cost of scale up for a basic package of reproductive health services – ranging from family planning, antenatal and delivery care to emergency obstetric care and HIV/STI prevention and treatment. The RHCTM consists of two main parts. The first part estimates the direct costs associated with providing an essential package of 45 reproductive health interventions. Interventions evaluated in the current RHCTM include: (1) family planning; (2) antenatal care, including treatment for chlamydia, gonorrhea and anemia; (3) abortion (incomplete and elective) and post-abortion complications; (4) delivery care; (5) emergency/pre-referral care; (6) assisted vaginal delivery (EmOC treatment of obstructed labor); (7) cesarean section; (8) postpartum hemorrhage; (9) puerperal sepsis; (10) severe pre-eclampsia/eclampsia; (11) treatment of long-term complications such as PID and obstetric fistula; and (12) postpartum care. The RHCTM also includes costs for additional maternal complications including: (1) premature rupture of membranes; (2) prolonged labor; (3) Trichomoniasis and (4) antepartum hemorrhage. The second part costs out activities and investment required to improve the health system of a country in order to scale up and provide the above package of reproductive health interventions. This includes investments in the physical and human infrastructure (building, rehabilitating, and equipping medical facilities; training and retaining staff; improving the referral and medical supply system) as well as demand creation, outreach, supervision, monitoring and evaluation activities. The RHCTM uses an ingredients approach to estimate the costs associated with an intervention. Each complication is associated with a drug, supplies, and personnel requirements for treatment. However, the estimate does not include costs associated with occupying a health facility bed or an outpatient visit; these costs were obtained from the WHO CHOICE database and Nigeria-specific estimates were used.

Most of the data in WHO CHOICE are from UN sources such as the UN Population Division, WHO's Global Burden of Disease and other databases, UNICEF's maternal health database, and Demographic and Health Surveys. The lists of drugs and supplies required to provide the interventions are based on WHO treatment guidelines. Costs are presented in 2005 US$, and drug prices are based on quotes from the UNICEF Supply Catalogue and the MSH International Drug Price Indicator. Personnel cost/salaries are based on information provided by WHO CHOICE. Salaries originally reported in 2000 I$ were converted to local currency units. These estimates were compared to estimates obtained from the International Labour Organization [ILO Laborsta], and the newest available estimates for personnel for each category were used.

| **Table 39:** Annual personnel costs by category (*2005 US$*) | | Model Input |
| --- | --- | --- |
|  | Auxiliary/Attendant | $3,836 |
|  | Nurse/Midwife | $5,533 |
|  | General Physician | $9,315 |
|  | Obstetrician | $14,710 |
|  | Paediatrician | $14,710 |
|  | Anaesthesist | $14,710 |
|  | Lab Technician | $3,836 |

The RHCTM does not include estimates of facility costs per case. For this, we drew on the Nigeria- specific estimates of unit costs for patient services provided in WHO-CHOICE. Since many interventions can occur outside a 20-minute visit time frame (e.g., 5 minutes or 30 minutes), we broke down the cost for outpatient visits according to an estimated cost per minute. In the following intervention-specific sections, we present cost estimates followed by tables outlining how these costs were derived from the RHCTM for the following intervention components: (1) drugs and supplies per case; (2) personnel costs per case; and (3) facility costs per case.

| **Table 40:** Costs of various interventions used in the model. | | | | |
| --- | --- | --- | --- | --- |
| **Service** | **Drugs and supplies** | **Personnel costs**  **per case** | **Facility charge**  **per case** | **Total cost**  **per case** |
| **Family planning** |  |  |  |  |
| Oral contraceptives | $5.09 | $1.68 | $6.77 | $13.54 |
| Injectable contraceptives | $3.85 | $1.92 | $7.74 | $13.51 |
| Condom (male) | $2.85 | $1.68 | $6.77 | $11.30 |
| Implants | $25.89 | $2.40 | $9.67 | $37.96 |
| IUD | $1.24 | $2.40 | $9.67 | $13.31 |
| Sterilization (female) | $4.73 | $6.42 | $12.13 | $23.28 |
| Sterilization (male) | $0.88 | $3.44 | $12.13 | $16.45 |
| **Pregnancy and delivery/abortion** |  |  |  |  |
| Prenatal care | $2.83 | $5.45 | $15.47; $16.82 | $23.75; $25.10 |
| Anemia prevention & treatment (based on severity) | $0.68; $1.02 |  |  | $0.68; $1.02 |
| Elective abortion |  |  |  | $21.87 |
| Incomplete abortion | $1.26 | $2.88 | $7.08 | $11.22 |
| Post abortion complications (bEmOC; cEmOC) | $12.03 | $8.24 | $28.31; $30.46 | $48.58 - $50.73 |
| Postnatal care | $0.23 | $1.44 | $5.80; $6.31 | $7.47; $7.98 |
| **Normal delivery** |  |  |  |  |
| At home (family) | $0.00 | $0.00 | $0.00 | $0.00 |
| At home (TBA; SBA) | $0.00 | $7.99 - $11.53 | $0.00 | $7.99 - $11.53 |
| At health center | $3.50 | $11.53 | $5.31 | $20.34 |
| At EmOC facility (bEmOC; cEmOC) | $3.67 | $11.53 | $3.54; $4.62 | $18.74; $18.82 |
| **Management of complications** |  |  |  |  |
| Eclampsia* at EmOC facility (bEmOC; cEmOC) | $7.20 | $17.07 | $49.54; $64.63 | $73.81; $88.91 |
| Eclampsia** at EmOC facility (bEmOC; cEmOC) | $7.20; $22.30 | $17.07; $29.24 | $49.54; $64.63 | $73.81; $116.17 |
| OL* at EmOC facility (bEmOC; cEmOC) | $6.00 | $10.55 | $7.08; $9.24 | $23.63; $25.79 |
| OL** at EmOC facility (bEmOC; cEmOC) | $6.00; $12.24 | $10.55; $33.09 | $7.08 ; $64.63 | $23.63; $109.96 |
| PPH* at EmOC facility (bEmOC; cEmOC) | $7.44 | $13.12 | $14.16; $18.48 | $34.72; $39.03 |
| PPH** at EmOC facility (bEmOC; cEmOC) | $7.44; $87.32 | $13.12; $26.53 | $14.16; $36.93 | $34.72; $150.78 |
| Sepsis* at EmOC facility (bEmOC; cEmOC) | $13.71 | $11.22 | $14.16; $18.48 | $39.09; $43.40 |
| Sepsis** at EmOC facility (bEmOC; cEmOC) | $13.71; $34.79 | $11.22; $12.17 | $14.16; $36.93 | $39.09; $83.90 |
| **Transportation** |  |  |  | $5.15 - $11.58 |
| IUD = intrauterine devices; TBA = traditional birth attendant; SBA = skilled birth attendant; bEmOC = basic emergency obstetric care; cEmOC = comprehensive emergency obstetric care; EmOC = emergency obstetric care; CS = Caesarean section; OL = obstructed labor; PPH = postpartum hemorrhage.  * represents serious but not life threatening complication;  ** represents a life threatening complication. | | | | |

Some of the assumptions made in determining the costs are described below. Contraceptives were assumed to be obtained at outpatient clinics/health posts. The cost of condoms does not cover those distributed by social marketing or community based distribution. Antenatal care costs reflects the cost of visits, including drugs, personnel as well as screening and treatment for STIs. It also includes urinalysis for glucose, ketones, pH, plus four visits, counseling and education (family planning, birth spacing, parenting, etc.) We added the cost of anemia treatment (iron and folate supplementation). However, we did not include the cost of anti-malaria interventions (IPT – intermittent preventive therapy; and ITN – insecticide treated nets). Incomplete abortions and post-abortal complications were assumed to be managed by manual vacuum aspiration, with or without treatment of sepsis or repair of genital tears.

We assume that for births that take place at home there are three possible levels of care: assistance by a family member, assistance by TBA, and assistance by SBA. For home deliveries, we include the cost of the attendant’s time but there are no facility charges, and no charges for drugs. Deliveries at a primary-level health post (i.e., sub center or health posts) utilize the following assumptions: all deliveries are attended by skilled staff, requires half a bed day, has mechanisms for referral to facility with EmOC; functions of bEmOC are not assumed to be present. For deliveries at a secondary-level health center (i.e., community health center or primary healthcare centers), basic EmOC is expected to be available. For deliveries at a tertiary-level facility (i.e., general hospitals, teaching hospitals, federal medical centers), comprehensive EmOC is expected to be available. To model the relationship between the costs of delivery estimated using the RHCTM tool ingredients-based approach for normal delivery and the costs of delivery at different levels of facilities, we applied a scaled factor to reflect the higher costs in a tertiary hospital versus secondary-level facility versus a birthing center or primary care health post. The scaling factor relied on the relative costs reported in the regional and country-specific WHO CHOICE databases for primary-, secondary- and tertiary- level facility bed-days and visits, as well as the relative costs of health provider salaries based on a distribution of increasingly skilled health providers comparing a primary-level birthing center or sub center to the tertiary-level hospital (e.g., specialists versus medical officers versus nurses). Results show that bEmOC facilities are 1.7 times (1.30 -1.97) more costly than primary-level health centers, posts or birthing centers; tertiary facilities are 2.25 (1.78-2.56) times more costly than primary-level facilities. Costs for transportation include the cost of transportation for a woman with a recognized complication that cannot be treated at the original birthing location (either true complication or false referral), and the cost for an attendant to accompany the woman during transport in some circumstances. There is a relative lack of data available on the cost of transportation in Nigeria from one birthing location (home, health post, bEmOC), to a higher-level facility (health post, bEmOC, cEmOC). We were able to leverage some data on transportation from other sectors to approximate increased costs associated with improving transport programs in rural regions. The range for transportation costs encompasses cost of transport from home to a health facility (birthing center, bEmOC or cEmOC facility), and between health facilities (birthing center to bEmOC or cEmOC and bEmOC to cEmOC). Using public access data, we also developed a transport cost calculator that incorporates information on distance, road density, cost of vehicle and fuel, and approximates a cost. Data used to develop this cost estimation tool were derived from various sources including the World Bank and national databases on road transport. This allowed for rough approximations for face validity using studies that reported mean distance (see **Table 27**). Management of obstructed labor at bEmOC facilities consists of assisted vaginal delivery with vacuum or forceps and Caesarean sections in cEmOC facilities. In addition to obstructed labor costs, we also included the cost of prolonged labor, which precedes the diagnosis of obstructed labor. Managing PPH in cEmOC facilities include services such as blood transfusion, advanced shock management, and/or surgery. The difference in the cost of management/treatment for PPH reflects primarily the lack of capacity to perform emergency transfusions at a bEmOC facility and increased personnel and facility charges at cEmOC facilities, while postpartum care includes a 30-minute visit by a skilled health provider and distribution of iron/folate supplementation.

**PART V: SUPPLEMENTAL RESULTS**

**Family planning**

**Table 41** shows the potential benefits of increasing the prevalence of modern contraceptives in the Northeast zone. Estimates from the National and Southwest zone analysis are contained in the manuscript. A complete reduction in the unmet need for contraception is predicted to reduce maternal deaths by about 13%. This could be increased over 43% if more than half of all women of reproductive age living in the Northeast zone use a modern contraceptive method.

| **Table 41:** Potential benefits that accompanied a stepwise reduction in the unmet need for contraception | | | | | | | | | | |
| --- | --- | --- | --- | --- | --- | --- | --- | --- | --- | --- |
|  |  |  | **Primary analysis of family planning** | | | | **Secondary analysis of family planning** | | | |
| **Analysis** | **Maternal Health Index** | **Status quo** | **Reducing the unmet need for contraception** | | | | **Increasing use of modern contraceptive method** | | | |
|  |  |  | **by 25%** | **by 50%** | **by 75%** | **by 100%** | **by 25%** | **by 30%** | **by 40%** | **by 50%** |
| **NE Zone** | Prevalence of modern methods of contraception (average) | 3.5% | 7.4% | 11.3% | 15.1% | 19.0% | 28.5% | 33.5% | 43.5% | 53.5% |
|  | Reduction in maternal deaths (%) | - | 3.2% | 6.3% | 9.5% | 12.7% | 20.7% | 25.0% | 33.7% | 42.7% |
|  | Total fertility rates | 7.30 | 7.03 | 6.80 | 6.56 | 6.33 | 5.75 | 5.44 | 4.80 | 4.16 |
|  | Lifetime risk of maternal deaths | 1 in 9 | 1 in 9 | 1 in 9 | 1 in 10 | 1 in 10 | 1 in 11 | 1 in 12 | 1 in 13 | 1 in 16 |
|  | Proportionate mortality risk | 33% | 32.2% | 31.4% | 30.7% | 29.8% | 27.7% | 26.5% | 24.1% | 21.4% |
|  | Maternal deaths averted per 100,000 | - | 354 | 712 | 1,064 | 1,428 | 2,328 | 2,810 | 3,791 | 4,795 |
|  | Additional costs per woman over lifetime (US$) | $0.00 | $0.63 | $1.26 | $1.87 | $2.51 | $4.08 | $4.91 | $6.59 | $8.29 |
|  | Additional cost to cohort over lifetime (million US$) | - | $2.48 | $4.97 | $7.41 | $9.93 | $16.12 | $19.41 | $26.06 | $32.79 |
|  | Cost effectiveness ratio (rounding) (US$ per YLS) | - | 5.8/YLS | 5.9/YLS | 5.9/YLS | 5.9/YLS | 6.0/YLS | 6.0/YLS | 6.0/YLS | 6.1/YLS |
| Each step is in comparison with current conditions. NE = Northeast. YLS = Year of life saved. In this analysis, the number of and quality of care in EmOC facilities as well as availability and access to safe abortion services remained unchanged. Each step is compared to status quo. | | | | | | | | | | |

**Upgrades**

**Table 42** shows the potential benefits derivable from increasing the number of and quality of care in EmOC facilities. In themselves, they are predicted to reduce maternal deaths by up to 60% national and in both zones being analyzed.

| **Table 42:** Potential benefits that accompanied increasing the number of and quality of care in EmOC facilities (termed “Upgrades”). | | | | | | |
| --- | --- | --- | --- | --- | --- | --- |
|  |  | **Upgrades** | | | | |
| **Analysis** | **Maternal Health Index** | **Current** | **Upgrade 1** | **Upgrade 2** | **Upgrade 3** | **Upgrade 4** |
| National | Reduction in maternal deaths (%) | 0.0% | 13.6% | 27.0% | 41.4% | 58.2% |
|  | Total Fertility rate | 5.93 | 5.95 | 5.98 | 6.02 | 6.06 |
|  | Lifetime risk of maternal deaths | 1 in 26 | 1 in 31 | 1 in 36 | 1 in 45 | 1 in 63 |
|  | Proportionate mortality risk | 14% | 12% | 10% | 8% | 6% |
|  | Maternal deaths averted in one year | 0 | 5,631 | 11,140 | 17,125 | 23,962 |
|  | Additional costs per woman over lifetime (US$) | $0.00 | $24.82 | $50.87 | $77.80 | $104.00 |
|  | Additional cost to cohort over lifetime (million US$) | - | $860.36 | $1,763.65 | $2,697.29 | $3,605.60 |
|  |  |  |  |  |  |  |
| Southwest zone | Reduction in maternal deaths (%) | 0.0% | 11.4% | 24.6% | 40.3% | 58.6% |
|  | Total Fertility rate | 4.60 | 4.60 | 4.61 | 4.62 | 4.62 |
|  | Lifetime risk of maternal deaths | 1 in 128 | 1 in 145 | 1 in 170 | 1 in 215 | 1 in 310 |
|  | Proportionate mortality risk | 3.1% | 2.7% | 2.3% | 1.9% | 1.3% |
|  | Maternal deaths averted in one year | 0 | 173 | 372 | 609 | 885 |
|  | Additional costs per woman over lifetime (US$) | $0.00 | $9.75 | $20.26 | $31.35 | $42.65 |
|  | Additional cost to cohort over lifetime (million US$) | - | $61.16 | $127.03 | $196.58 | $267.49 |
|  |  |  |  |  |  |  |
|  |  | **Current** | **Upgrade 2** | **Upgrade 4** | **Upgrade 6** | **Upgrade 8** |
| Northeast zone | Reduction in maternal deaths (%) | 0.0% | 15% | 29% | 44% | 60% |
|  | Total Fertility rate | 7.23 | 7.35 | 7.45 | 7.55 | 7.67 |
|  | Lifetime risk of maternal deaths | 1 in 9 | 1 in 10 | 1 in 13 | 1 in 16 | 1 in 22 |
|  | Proportionate mortality ratio | 33% | 29% | 25% | 21% | 16% |
|  | Maternal deaths averted in one year | 0 | 2,143 | 4,176 | 6,259 | 8,501 |
|  | Additional costs per woman over lifetime (US$) | $0.00 | $42.18 | $88.05 | $137.12 | $185.71 |
|  | Additional cost to cohort over lifetime (million US$) | - | $166.80 | $348.20 | $542.22 | $734.37 |
| For this analysis, the prevalence of use of modern contraceptives as well as the availability and access to safe abortion services remain unchanged. Each step is compared to status quo. | | | | | | |

**Safe abortion**

**Table 43** shows the potential benefits obtained from increasing availability and access to safe abortion services. Universal access is predicted to reduce maternal deaths by up to 3% Nation wide, and 8% in the Southwest zone. However, it could result in an increased number of maternal deaths in the Northeast zone. A possible explanation for this is that with increasing access to safe abortion services, women who would otherwise have been rendered infertile following an unsafe abortion would be spared, and join the pool of women who face risks of maternal deaths. However, this phenomenon was observed only when availability and access to safe abortion services was increased in isolation. The reverse was the case when it was combined with increased family planning use and upgrades.

| **Table 43:** Potential benefits that accompanied increasing availability and access to safe abortion services. | | | | | | |
| --- | --- | --- | --- | --- | --- | --- |
|  |  | **Stepwise increase in access to safe abortion services** | | | | |
| **Analysis** | **Maternal Health Index** | **Current** | **25%** | **50%** | **75%** | **100%** |
| National | Reduction in maternal deaths (%) | 0% | 0.8% | 1.5% | 2.3% | 3.1% |
|  | Total Fertility rates | 5.93 | 5.97 | 6.03 | 6.07 | 6.14 |
|  | Lifetime risk of maternal death | 1 in 26 | 1 in 27 | 1 in 27 | 1 in 27 | 1 in 27 |
|  | Proportionate mortality risk | 14% | 13% | 13% | 13% | 13% |
|  | Maternal deaths averted in one year | 0 | 398 | 802 | 1,211 | 1,626 |
|  | Additional costs per woman over lifetime (US$) | $0.00 | -$1.70 | -$3.43 | -$5.19 | -$6.97 |
|  | Additional cost to cohort over lifetime (million US$) | $0.00 | -$59.05 | -$119.00 | -$179.87 | -$241.67 |
|  |  |  |  |  |  |  |
| Northeast zone | Reduction in maternal deaths (%) | 0% | -0.38% | -0.77% | -1.15% | -1.54% |
|  | Total Fertility rates | 7.23 | 7.32 | 7.38 | 7.46 | 7.54 |
|  | Lifetime risk of maternal death | 1 in 9 | 1 in 9 | 1 in 9 | 1 in 9 | 1 in 9 |
|  | Proportionate mortality risk | 33% | 33% | 33% | 33% | 33% |
|  | Maternal deaths averted in one year | 0 | -14 | -28 | -42 | -56 |
|  | Additional costs per woman over lifetime (US$) | $0.00 | -$2.22 | -$4.48 | -$6.78 | -$9.13 |
|  | Additional cost to cohort over lifetime (million US$) | $0.00 | -$8.77 | -$17.71 | -$26.82 | -$36.10 |
|  |  |  |  |  |  |  |
|  |  | **Current** | **70%** | **80%** | **90%** | **100%** |
| Southwest zone | Reduction in maternal deaths (%) | 0% | 2.1% | 4.1% | 6.2% | 8.3% |
|  | Total Fertility rates | 4.60 | 4.60 | 4.62 | 4.63 | 4.63 |
|  | Lifetime risk of maternal death | 1 in 128 | 1 in 131 | 1 in 134 | 1 in 137 | 1 in 140 |
|  | Proportionate mortality ratio | 3.1% | 3.0% | 2.9% | 2.9% | 2.8% |
|  | Maternal deaths averted in one year | 0 | 32 | 65 | 97 | 130 |
|  | Additional costs per woman over lifetime (US$) | $0.00 | -$0.43 | -$0.86 | -$1.29 | -$1.72 |
|  | Additional cost to cohort over lifetime (million US$) | $0.00 | -$2.67 | -$5.36 | -$8.06 | -$10.78 |
| For this analysis, the prevalence of use of modern contraceptives as well as the prevalence of use of modern contraceptives remain unchanged. Each step is compared to status quo. | | | | | | |

**Integrated package of interventions**

**Table 44** shows the potential benefits from applying all three interventions in various combinations. It applies only to the national analysis, and with full coverage, it predicts a 70% reduction in maternal deaths.

| **Table 44:** Percentage reduction in maternal deaths when interventions were simultaneously applied in various combinations nationwide. | | | | | |
| --- | --- | --- | --- | --- | --- |
|  | **Increasing availability of safe abortion** | | | | |
|  | **Current** | **25%** | **50%** | **75%** | **100%** |
| **Base Case** |  |  |  |  |  |
| -with no change in family planning | 0.0% | 0.8% | 1.5% | 2.3% | 3.1% |
| -with family planning increased by 25% of unmet need | 5.9% | 6.6% | 7.4% | 8.2% | 9.0% |
| -with family planning increased by 50% of unmet need | 9.7% | 10.4% | 11.2% | 12.0% | 12.8% |
| -with family planning increased by 75% of unmet need | 13.5% | 14.3% | 15.1% | 15.8% | 16.6% |
| -with family planning increased by 100% of unmet need | 17.4% | 18.2% | 18.9% | 19.7% | 20.5% |
| **Upgrade 1** |  |  |  |  |  |
| -with no change in family planning | 13.6% | 14.5% | 15.4% | 16.3% | 17.2% |
| -with family planning increased by 25% of unmet need | 18.7% | 19.6% | 20.4% | 21.3% | 22.2% |
| -with family planning increased by 50% of unmet need | 22.0% | 22.9% | 23.7% | 24.6% | 25.5% |
| -with family planning increased by 75% of unmet need | 25.4% | 26.2% | 27.0% | 27.9% | 28.8% |
| -with family planning increased by 100% of unmet need | 28.7% | 29.5% | 30.4% | 31.2% | 32.1% |
| **Upgrade 2** |  |  |  |  |  |
| -with no change in family planning | 27.0% | 28.0% | 29.0% | 30.0% | 31.0% |
| -with family planning increased by 25% of unmet need | 31.3% | 32.2% | 33.2% | 34.2% | 35.2% |
| -with family planning increased by 50% of unmet need | 34.1% | 35.1% | 36.0% | 37.0% | 38.0% |
| -with family planning increased by 75% of unmet need | 36.9% | 37.9% | 38.8% | 39.7% | 40.7% |
| -with family planning increased by 100% of unmet need | 39.8% | 40.7% | 41.6% | 42.5% | 43.4% |
| **Upgrade 3** |  |  |  |  |  |
| -with no change in family planning | 41.5% | 42.6% | 43.8% | 44.9% | 46.1% |
| -with family planning increased by 25% of unmet need | 45.0% | 46.1% | 47.2% | 48.2% | 49.4% |
| -with family planning increased by 50% of unmet need | 47.3% | 48.3% | 49.4% | 50.4% | 51.5% |
| -with family planning increased by 75% of unmet need | 49.5% | 50.6% | 51.6% | 52.6% | 53.7% |
| -with family planning increased by 100% of unmet need | 51.8% | 52.8% | 53.8% | 54.8% | 55.8% |
| **Upgrade 4** |  |  |  |  |  |
| -with no change in family planning | 58.2% | 59.5% | 60.7% | 62.0% | 63.3% |
| -with family planning increased by 25% of unmet need | 60.7% | 61.9% | 63.1% | 64.3% | 65.6% |
| -with family planning increased by 50% of unmet need | 62.3% | 63.5% | 64.6% | 65.8% | 67.0% |
| -with family planning increased by 75% of unmet need | 64.0% | 65.1% | 66.2% | 67.3% | 68.5% |
| -with family planning increased by 100% of unmet need | 65.6% | 66.7% | 67.8% | 68.9% | 70.0% |

In determining the potential benefits of scaling up all three interventions over a 12 year period, we assumed that only women aged 15 – 45 years would benefit from this programs, and started out by selecting these group of women from the 2008 UN population projection for Nigerian women. It is a table that contains the projected population of women aged 0 – 99 years from 1995 to 2110. In this table, survivors from any age group (e.g. 15 year old women in 2010) were carried forward into the subsequent age group for the following year (i.e. 16 year olds in 2011).

Our first focus was on women aged 15 – 45 years in year 2006 and 2007. To this cohort, we applied model generated age-specific annual probabilities of death and proportionate mortality risks for the base case. Our population projections for this group of women approximated UN projections (with age-specific variations in population ranging between -0.7% to 0.1% of UN data), as did our estimate of the number of maternal deaths each year (48,483 and 49,810 in 2007 and 2008 respectively). Next, we focused on a 13-year period, from 2010 to 2022. The starting population for this hypothetical cohort was made up of women aged 15 – 45 years in 2010. In subsequent years, women entered the cohort when whey turned 15 years old, and left the cohort when they turned 46 years old. Assuming there was no change in the coverage of any intervention over the next 12 years, we applied model generated age-specific annual probabilities of death, proportionate mortality risks, and costs per case for the base case to the starting populations, survivors and women entering the cohort over the 12 year period. We determined that over 740,000 maternal deaths could occur over this period (**Table 45)**.

| **Table 45:** Projected number of maternal deaths and costs to the cohort each year from 2011 – 2022. | | | | | | | | | | | | | |
| --- | --- | --- | --- | --- | --- | --- | --- | --- | --- | --- | --- | --- | --- |
|  | 2011 | 2012 | 2013 | 2014 | 2015 | 2016 | 2017 | 2018 | 2019 | 2020 | 2021 | 2022 | Grand Total |
| Maternal deaths | 53,704 | 55,135 | 56,592 | 58,071 | 59,568 | 61,081 | 62,612 | 64,162 | 65,725 | 67,294 | 68,864 | 70,433 | 743,240 |
| Costs (million US$) | $246.40 | $252.76 | $259.23 | $265.81 | $272.51 | $279.30 | $286.19 | $293.18 | $300.25 | $307.40 | $314.58 | $321.77 | $3,399.38 |
| This is under the assumption that there is no change to the current coverage of interventions. | | | | | | | | | | | | | |

We then considered the three approaches mentioned in the manuscript, and applied them in the order shown in **Table 5** therein. In a similar manner described above, we applied model generated age-specific annual probabilities of death, proportionate mortality risks, and costs per case for the various coverage and combinations of interventions to the starting populations, survivors, and women entering the cohort over the 12-year period. The results are shown in **Table 47.**

| **Table 47:** Projected number of maternal deaths and costs to the cohort with the various approaches each year from 2011 – 2022. | | | | | | | | | | | | | | |
| --- | --- | --- | --- | --- | --- | --- | --- | --- | --- | --- | --- | --- | --- | --- |
|  |  | 2011 | 2012 | 2013 | 2014 | 2015 | 2016 | 2017 | 2018 | 2019 | 2020 | 2021 | 2022 | Grand Total |
| Approach 1 | Maternal deaths | 50,909 | 50,223 | 49,218 | 48,380 | 42,598 | 43,687 | 37,487 | 38,426 | 30,948 | 31,700 | 22,225 | 22,744 | 468,545 |
|  | Costs (million US$) | $250.28 | $260.67 | $271.39 | $282.46 | $327.78 | $336.03 | $386.60 | $396.18 | $451.73 | $462.70 | $520.46 | $532.66 | $4,478.93 |
|  |  |  |  |  |  |  |  |  |  |  |  |  |  |  |
| Approach 2 | Maternal deaths | 46,601 | 47,851 | 41,726 | 42,829 | 35,385 | 36,300 | 26,796 | 27,475 | 25,959 | 25,052 | 23,740 | 22,762 | 402,475 |
|  | Costs (million US$) | $287.58 | $295.04 | $348.30 | $357.26 | $416.14 | $426.70 | $488.36 | $500.56 | $506.03 | $515.16 | $524.19 | $533.07 | $5,198.38 |
|  |  |  |  |  |  |  |  |  |  |  |  |  |  |  |
| Approach 3 | Maternal deaths | 44,040 | 45,220 | 37,539 | 38,531 | 29,588 | 30,352 | 20,198 | 20,709 | 21,224 | 21,742 | 22,260 | 22,778 | 354,180 |
|  | Costs (million US$) | $289.27 | $296.78 | $348.43 | $357.38 | $410.74 | $421.17 | $473.16 | $484.99 | $496.98 | $509.09 | $521.25 | $533.42 | $5,142.64 |

The differences in projected maternal deaths and costs to the cohort are contained in **Table 5** of the manuscript.

**Uncertainty**

To assess for uncertainty, we varied several model inputs to limits suggested by empiric studies. These inputs include the incidence and case fatality rates of direct maternal complications, as well as the effectiveness and cost of maternal interventions (cost were varied between 50% and 100% of the original inputs). While varying these inputs, we improved the coverage of effective interventions (i.e. we reduced the unmet need for contraception, increased access to safe abortion and post-abortion care, as well as access to optimal intrapartum and postpartum care). In all instances, the increasing availability of these interventions were cost effective (with ICERs less than $550 per YLS).

Although the cost-effectiveness (CE) ratios, the ICERs and the predicted reduction in maternal deaths were largely robust, varying the incidence or CFR of direct maternal complications resulted in outcomes that did not meet calibration targets. For example, increasing the CFR led to over 130,000 maternal deaths annually, almost three times what is reported in available literature. The outcomes from varied effectiveness of interventions were close to calibration targets because of low coverage of maternal services.

**Table 48:** Assessing the effect of uncertainty in some input parameters

| **Analysis** | **Predicted number of maternal deaths** | **Maternal mortality ratio (MMR) per 100,000 live births** | **Total fertility rate (TFR)** | **Lifetime risk of maternal death** | **Proportionate mortality risk** |
| --- | --- | --- | --- | --- | --- |
|  |  |  |  |  |  |
| **Data from literature (i.e. calibration targets) [3, 4, 112]** | 50,000 | 800 | 5.7 | 1 in 23 | 18% |
|  |  |  |  |  |  |
| **Data from analysis** |  |  |  |  |  |
| - Unchanged parameters (i.e. incidences, CFR and effectiveness) | 53,000 | 800 | 5.8 | 1 in 26 | 13% |
| - Reduced incidences of direct maternal complications | 36,689 | 536 | 5.9 | 1 in 40 | 9% |
| - Increased incidence of direct maternal complications | 85,888 | 1,340 | 5.7 | 1 in 16 | 20% |
| - Reduced CFR of direct maternal complications | 37,625 | 550 | 5.9 | 1 in 39 | 10% |
| - Increased CFR of direct maternal complications | 135,598 | 2,240 | 5.6 | 1 in 10 | 30% |
| - Reduced effectiveness of maternal interventions | 53,389 | 800 | 5.8 | 1 in 27 | 13% |
| - Increased effectiveness of maternal interventions | 53,000 | 800 | 5.8 | 1 in 27 | 13% |

**PART VI: REFERENCES:**
